# Interpretation of the past, present, and future of organoid technology: an updated bibliometric analysis from 2009 to 2024

**DOI:** 10.3389/fcell.2024.1433111

**Published:** 2024-08-13

**Authors:** Baozhen Qu, Qiang Mu, Huanhuan Bi, Yuxian Chen, Qitang Wang, Xuezhen Ma, Linlin Lu

**Affiliations:** ^1^ Qingdao Cancer Prevention and Treatment Research Institute, Qingdao Central Hospital, University of Health and Rehabilitation Sciences (Qingdao Central Hospital), Qingdao, China; ^2^ The First Department of Breast Surgery, Qingdao Central Hospital, University of Health and Rehabilitation Sciences (Qingdao Central Hospital), Qingdao, China; ^3^ College of Medicine, Qingdao University, Qingdao, China; ^4^ Department of Oncology, Qingdao Central Hospital, University of Health and Rehabilitation Sciences (Qingdao Central Hospital), Qingdao, China

**Keywords:** organoid, 3D cultures, drug screening, drug development, precision medicine, cancer research, hotspots, visualization

## Abstract

Organoid technology has been developed rapidly in the past decade, which involves the exploration of the mechanism of development, regeneration and various diseases, and intersects among multiple disciplines. Thousands of literature on 3D-culture or organoids have been published in the research areas of cell biology tissue engineering, nanoscience, oncology and so on, resulting in it being challenging for researchers to timely summarize these studies. Bibliometric statistics is a helpful way to help researchers clarify the above issues efficiently and manage the whole landscape systematically. In our study, all original articles on organoids were included in the Web of Science database from January 2009 to May 2024, and related information was collected and analyzed using Excel software, “bibliometrix” packages of the R software, VOSviewer and CiteSpace. As results, a total of 6222 papers were included to classify the status quo of the organoids and predict future research areas. Our findings highlight a growing trend in publications related to organoids, with the United States and Netherlands leading in this field. The University of California System, Harvard University, Utrecht University and Utrecht University Medical Center have emerged as pivotal contributors and the key authors in the field include Clevers, H, Beekman, JM and Spence JR. Our results also revealed that the research hotspots and trends of organoids mainly focused on clinical treatment, drug screening, and the application of materials and technologies such as “hydrogel” and “microfluidic technology” in organoids. Next, we had an in-depth interpretation of the development process of organoid research area, including the emergence of technology, the translation from bench to bedsides, the profiles of the most widely studied types of organoids, the application of materials and technologies, and the emerging organoid-immune co-cultures trends. Furthermore, we also discussed the pitfalls, challenges and prospects of organoid technology. In conclusion, this study provides readers straightforward and convenient access to the organoid research field.

## 1 Introduction

Currently, accumulated studies have shown organoids are powerful model systems that facilitate our understanding of development and regeneration, health and disease (Sachs et al., 2019). During the past 10 years, organoids have been generated from numerous healthy and diseased tissues and utilized for a broad range of applications in biological, medical and translational research, especially in rare disease modeling, gene editing, drug screening (Driehuis et al., 2020) and precision therapy (Schutgens and Clevers, 2020). Indeed, cell and animal models have provided important insights into the development, pathogenesis, and treatment of a variety of diseases over the last century. However, it is difficult to reproduce the key aspects of human organs, especially their complex structure and metabolic function, since cell and animal models have elementary differences in human genetics, physiology, immunology, and the mechanisms of different disease progressions. Specifically, two-dimensional (2D) cell lines cannot reconstruct complex cell-cell and cell-matrix interactions, gene mutations, chromosome abnormalities, and microenvironments that are presented in human tissues (Bein et al., 2018). Species differences between animal models and humans are the fundamental barriers to the use of animal models. Now, organoids provide a new perspective to overcome these challenges mentioned above.

In 1946, the “organoid” term was employed for the first time to define a tumor-derived mass isolated from a human tissue. Subsequently, all tissue masses resultant from transplants were defined as organoids (Silva et al., 2019). Since the 1980s, three-dimensional (3D) cultures have been recognized as organoids in the scientific community (Abraham et al., 2024). Until 2009, Dutch scientist Professor Clevers, H discovered that the single leucine-rich repeat containing G protein-coupled receptor 5 (Lgr5) expressing adult intestinal stem cells could form 3D intestinal organoids in matrigel that self-organize and differentiate into crypt-villus structures (Sato et al., 2009). This was the first report on establishing 3D organoid culture derived from a single adult stem cell, which opened a new era of development in organoid technology. In 2014, organoids were defined as organ-like structures formed by stem cells through the development of a 3D culture system (Lancaster and Knoblich, 2014). Subsequently, the culture of other human organoids was based on the culture protocols of intestinal organoids, for example, liver (Huch et al., 2015; Hu et al., 2018) lung (Sachs et al., 2019), pancreas (Boj et al., 2015), prostate (Karthaus et al., 2014), stomach (Bartfeld et al., 2015), fallopian tube (Kessler et al., 2015), taste buds (Ren et al., 2014), bladder (Mullenders et al., 2019), breast (Sachs et al., 2018), salivary glands (Maimets et al., 2016), esophagus (DeWard et al., 2014) have been established successfully. Compared to 2D cell lines, organoids can not only maintain their capability of self-renewing *in vitro*, but also retain their original characteristics *in vivo*. Tumor organoids can hold their original histopathological features after xenotransplantation in immunodeficient mice, which means that advanced experimental studies can be conducted at the organoid level, such as biomarker screening, live imaging, targeted sequencing, whole exome sequencing (Roerink et al., 2018), even gene editing technology (Artegiani et al., 2020). Organoids also play an important role in clinical research. Organoid disease models include but are not limited to intestinal diseases (Gjorevski et al., 2016), brain diseases (Velasco et al., 2019), and heart diseases (Scherba et al., 2022). At present, a significant number of scientific research related to the above topics is contributed by worldwide countries and authors, resulting in challenges for researchers to timely summarize this research, objectively evaluate the most meaningful research or clarify the trends of the study hotspots. Bibliometrics is a new data-driven method that applies statistical methods to scientific outputs, which has knowledge-oriented quantitative functions. It can find out the knowledge association between publications through the filtering and processing of massive information, therefore digging out the potential knowledge value. Bibliometric statistics is a useful way to help researchers clarify the above issues efficiently and manage the whole landscape systematically. Bibliometric methods have been used to explore the academic achievements of researchers, institutions and countries in specific research areas, current trends, and the superiority of each territory.

There are several published bibliometric articles related to organoids but with limitations to some extent. These recently published bibliometric analyses mainly focused on subdivided organoids research areas respectively, including intestinal organoids (Zhang et al., 2021), cerebral organoids (Ding et al., 2022), and systematic comparison of organoid and organ-on-a-chip (Wang et al., 2020). A recent study by Li et al. described the characteristics of global human organoid research with a retrieval time span of 1 January 2003 to 10 January 2024 through bibliometric analysis (Li et al., 2024). Compared with this study, our research performed a retrieval and statistical analysis for original articles from March 2009 (the opening of the new era of development in organoid technology) to May 2024 (the latest update time). Besides, compared with the paper by Li et al. (Li et al., 2024) that only used “organoid” as the retrieval strategy, we designed a retrieval strategy [TI=(((organoid) OR (organoids)) OR (((3D) OR (three dimensions) OR (3 dimensions) OR (three dimensional) OR (3 dimensional)) AND (cell OR tissue) AND ((culture) OR (cultures) OR (cultured)))) AND DT= (Article) NOT DT=((Book Chapter) OR (Data Paper)) NOT TI=((guideline) OR (consensus) OR (meta-analyses) OR (meta-analysis) OR (meta-analysis) OR (data pooling) OR (overview) OR (current status) OR (review) OR (progress to date))] that considered various writing formats and conducted multiple tests. The above two points eliminated redundant information interference and ensured the accuracy and timeliness of the articles we retrieved for subsequent bibliometric analysis. More importantly, with the help of bibliometrics, a systematic and macroscopic research tool, we have an in-depth interpretation of the development process of the field of organoid research, including the background and reasons for the emergence of technology, the development of this technology in the laboratory and the extension from laboratory to clinical application, the progress of the most widely studied types of organoids, the application of materials and technologies in organoids, and the role of organoids in emerging immunotherapy. Finally, the existing limitations and future development prospects are analyzed.

## 2 Methods

### 2.1 Study selection

Science Citation Indexing Expanded database from the Web of Science Core Collection (WoSCC) was selected to identify the papers on organoids. It is the most frequently adopted and widely accepted database for bibliometric analyses, containing almost all excellent and reliable original articles and providing overall citation index records. The author designed a retrieval strategy that considered various writing formats and conducted multiple tests to ensure its accuracy. After that, the scientific output data downloaded from the Science Citation Indexing Expanded database was retrieved on May 2024, with a retrieval time span of 2009–2024 and the publication type limited to articles and reviews in English. Furthermore, the raw data including title, abstract, keywords, author, publication date, journal name, country/region, institution, and citations were extracted from the Web of Science for subsequent statistical analysis. To evaluate the most influential in the field, we also selected the 100 most cited studies and defined them as top papers for analysis. Then, the authors identified the journals that published top papers and calculated the top cited paper rates (TPR) of the journals (percentage of top papers among all papers in the field of organoids in a journal). Journals with a TPR >10% were considered as major journals on organoids (Liu et al., 2022).

### 2.2 Statistical analysis

Descriptive statistical analysis and graphical analysis were performed by Excel software (Microsoft, Redmond, WA, USA). Bibliometric analysis and data visualization were conducted using the “bibliometrix” packages of the R software and the software VOSviewer (v1.6.14). VOSviewer can generate three different types of visual maps: network, density, and overlay, each with a unique meaning (van Eck and Waltman, 2010). In these knowledge maps, each node represents an element including a country, institution, or author. The links between nodes describe the relationships between these elements, and the size of nodes which is represented by the total link strength (TLS) can be determined by multiple factors, such as the number of publications and the frequency of citations or appearances. The nodes and links are color-coded to distinguish clusters or to indicate the corresponding average appearing year (van Eck and Waltman, 2017). In this article, we established a literature co-occurrence network based on journals, countries, authors, and keywords via VOSviewer, and conducted co-occurrence analysis and visualization.

CiteSpace is a Java-based bibliometric tool that provides another avenue for analyzing the evolution and research clusters within a given topic (Chen, 2004). In this study, we used CiteSpace (v6.2. R3) to conduct visual analyses including burst words analysis and reference co-citation analysis. These analyses enable us to identify references or keywords that have received significant attention during a specific time period, a process also known as burst detection. The parameter settings for CiteSpace in this study were as follows: time span (from January 2009 to May 2024), slice length (3 years), selection criterion (g-index with k = 25), link retention factor (LRF), lookback years (LBY), and pruning method.

In summary, we first conducted an academic contribution evaluation using the “bibliometrix” packages of the R software, then used software VOSviewer to conduct co-occurrence analysis to evaluate the research status in the field of organoids, and finally evaluated the research hotspots and development trends in the field of organoids by using CiteSpace software.

## 3 Results

### 3.1 General analysis

**FIGURE 1:**
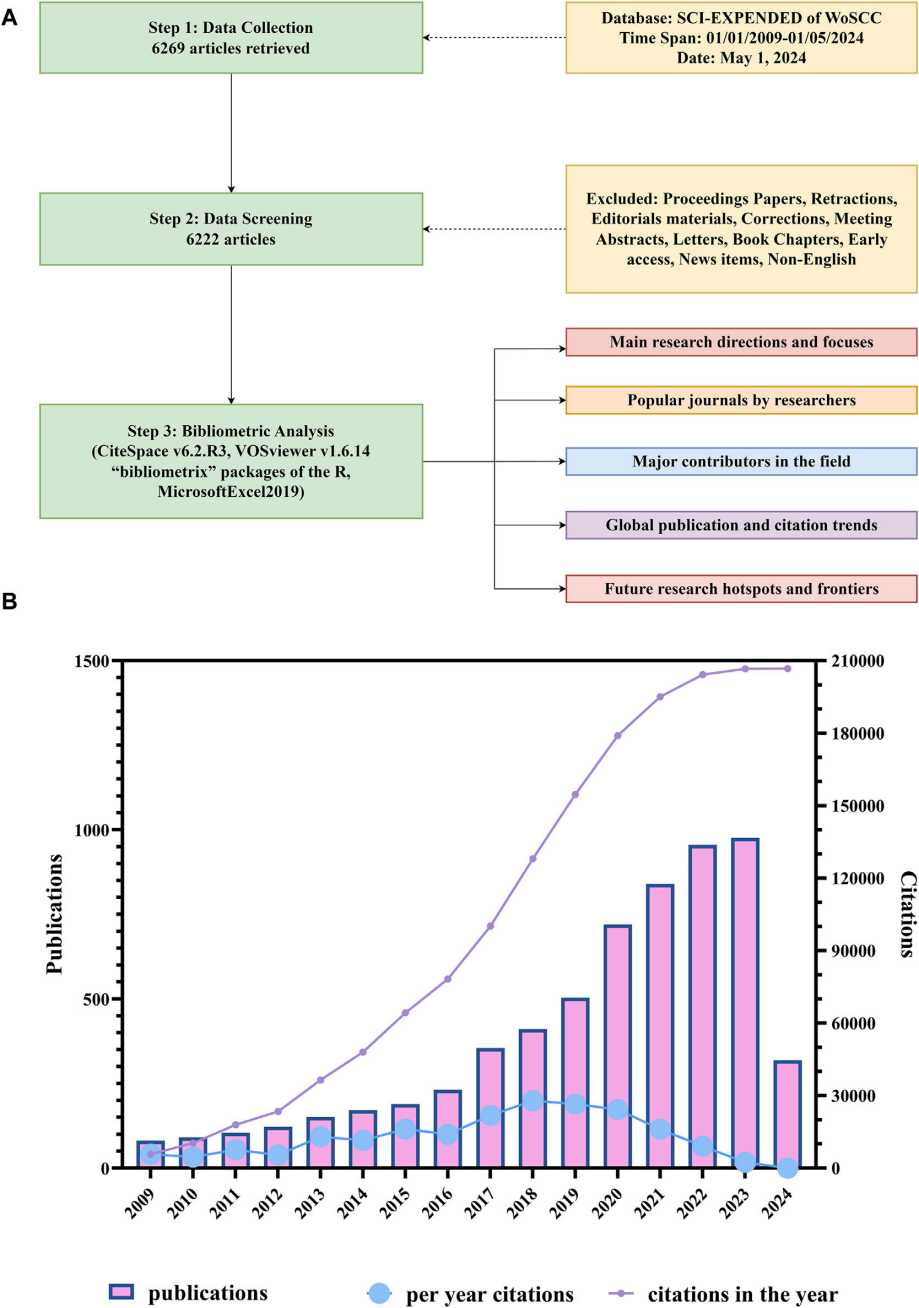
Flowchart of literature screening and time of publication and distribution of citations of the papers on organoids. **(A)** Flowchart of literature screening and data analysis. **(B)** Time of publication and distribution of citations of the papers on organoids. The blue line indicates the average citations of papers published each year. The purple line indicates the total citations of all papers published in the same year.

The specific retrieval process is shown in Figure 1A and the literature search yielded 6,222 papers on organoids published since 2009. As shown in Figure 1B, the number of publications is growing year by year, but the rise in 2017 was the most significant (53%). In terms of citations, the total citation number of these 6,222 papers was 206724 of which papers published in 2018 (27936/206724) contributed the greatest citation number. Furthermore, articles published in 2013 had the highest average citation number per paper (86.4).

Supplementary Table S1 listed the identified 100 most cited articles, which accounted for only 1.6% of the total number of publications but their citation number accounted for 27.4% of citations for all articles on organoids (56740/206724). The median number of citations of the top papers was 425.5 (range: 278–3035). The top 10 cited papers were listed in Table 1. The paper titled “Cerebral organoids model human brain development and microcephaly” by Lancaster et al. (2013) published in *Nature* had the highest number of total (3035) and average citations per year (286.77). In the past decade, it has been found that the number of cited articles on brain organoids ranked first, highlighting the important position of neuroscience in the academic community (See the text 3.6). This study opened a new era in the field of brain development and disease research. In this study, researchers successfully cultured a human pluripotent stem cell (PSC)-derived cerebral organoid for the first time, which recapitulated features of human cortical developments. This method not only summarized the basic mechanisms of mammalian neural development, but also demonstrated the characteristics of human brain development. More importantly, researchers have constructed a brain organoid model of microcephaly and simulated certain aspects of microcephaly, providing new insights into the pathogenesis of brain diseases. The paper with the title “Long-term expansion of epithelial organoids from human colon, adenoma, adenocarcinoma, and Barrett’s epithelium” (Sato et al., 2011) had the second highest number of total (2,397) and average citations per year (193.05). In this article, Sato, T and colleagues established long-term expansion of epithelial organoids from human colon, adenoma, adenocarcinoma, and Barrett’s epithelium. These cultures had no inherent limitations on the replication potential of adult stem cells and could be used to study infections, inflammation, or tumor tissues from the human intestinal tract, and might be applied in regenerative biology through *ex vivo* amplification of intestinal epithelium. This study has become the cornerstone for future researchers to use organoid technology to study intestinal diseases. The paper titled “Hydrogels as extracellular matrix mimics for 3D cell culture” published by Tibbitt and Anseth (2009) ranked third in total citations (1959). In this research, the authors discussed the use of synthetic and natural hydrogels as scaffolds for 3D cell culture, and synthetic hydrogels combined with complex biochemical and mechanical cues as mimics of natural extracellular matrix. They pointed out that the continuous progress of synthetic-biologic hydrogel hybrids was needed to provide a powerful platform for investigating cell physiology and fabricating tissue outside of the organism, which provided a new perspective for the advancement of organoid culture technology in the future. Among the top 10 cited articles, the journal *cell* holed the largest number of publications. In terms of research focus, eight out of these articles related to disease research, with six of them specifically associating with topics of cancer. The remaining two articles discussed advancements in organoid culture methods and the application of new technologies in organoids. The paper titled “Identification of SARS-CoV-2 inhibitors using lung and colonic organoids” by Han et al. (Han et al., 2021) published in *Nature* was the most recent publication among the 100 most cited papers. This study emphasized that lung and colon organoids derived from human PSCs infected with SARS-CoV-2 could serve as disease models for studying SARS-CoV-2 infection and provided valuable resources for drug screening to identify candidate COVID-19 therapies.

**TABLE 1:** The 10 most cited papers in organoids between 2009 and 2024.

Rank	Title	Corresponding Author	Journal	Year	Total citations	Average citations per year
1	Cerebral organoids model human brain development and microcephaly	Knoblich, JA	Nature	2013	3035	286.77
2	Long-term Expansion of Epithelial Organoids From Human Colon, Adenoma, Adenocarcinoma, and Barrett’s Epithelium	Sato, T	Gastroenterology	2011	2,397	193.05
3	Hydrogels as Extracellular Matrix Mimics for 3D Cell Culture	Anseth, KS	Biotechnology and Bioengineering	2009	1959	132.81
4	Prospective Derivation of a Living Organoid Biobank of Colorectal Cancer Patients	Garnett, MJ	Cell	2015	1,472	165.08
5	Organoid Models of Human and Mouse Ductal Pancreatic Cancer	Clevers, H	Cell	2015	1,363	147.35
6	Brain-Region-Specific Organoids Using Mini-bioreactors for Modeling ZIKV Exposure	Song, HJ; Ming, GL	Cell	2016	1,343	169.64
7	Patient-derived organoids model treatment response of metastatic gastrointestinal cancers	Valeri, N	Science	2018	1,059	171.73
8	A Living Biobank of Breast Cancer Organoids Captures Disease Heterogeneity	Clevers, H	Cell	2018	1,029	164.64
9	Organoid Cultures Derived from Patients with Advanced Prostate Cancer	Chen, Y	Cell	2014	1,015	105.91
10	Functional Repair of CFTR by CRISPR/Cas9 in Intestinal Stem Cell Organoids of Cystic Fibrosis Patients	Clevers, H	Cell Stem Cell	2013	957	92.61

### 3.2 Journals

The authors then identified the journals which published top papers and calculated the TPR of the journals. The time span extended from 2009 to 2024. Journals with a TPR >10% were considered major journals on organoids. The papers published in these journals since 2021 were analyzed to evaluate recent research hotspots.

**FIGURE 2:**
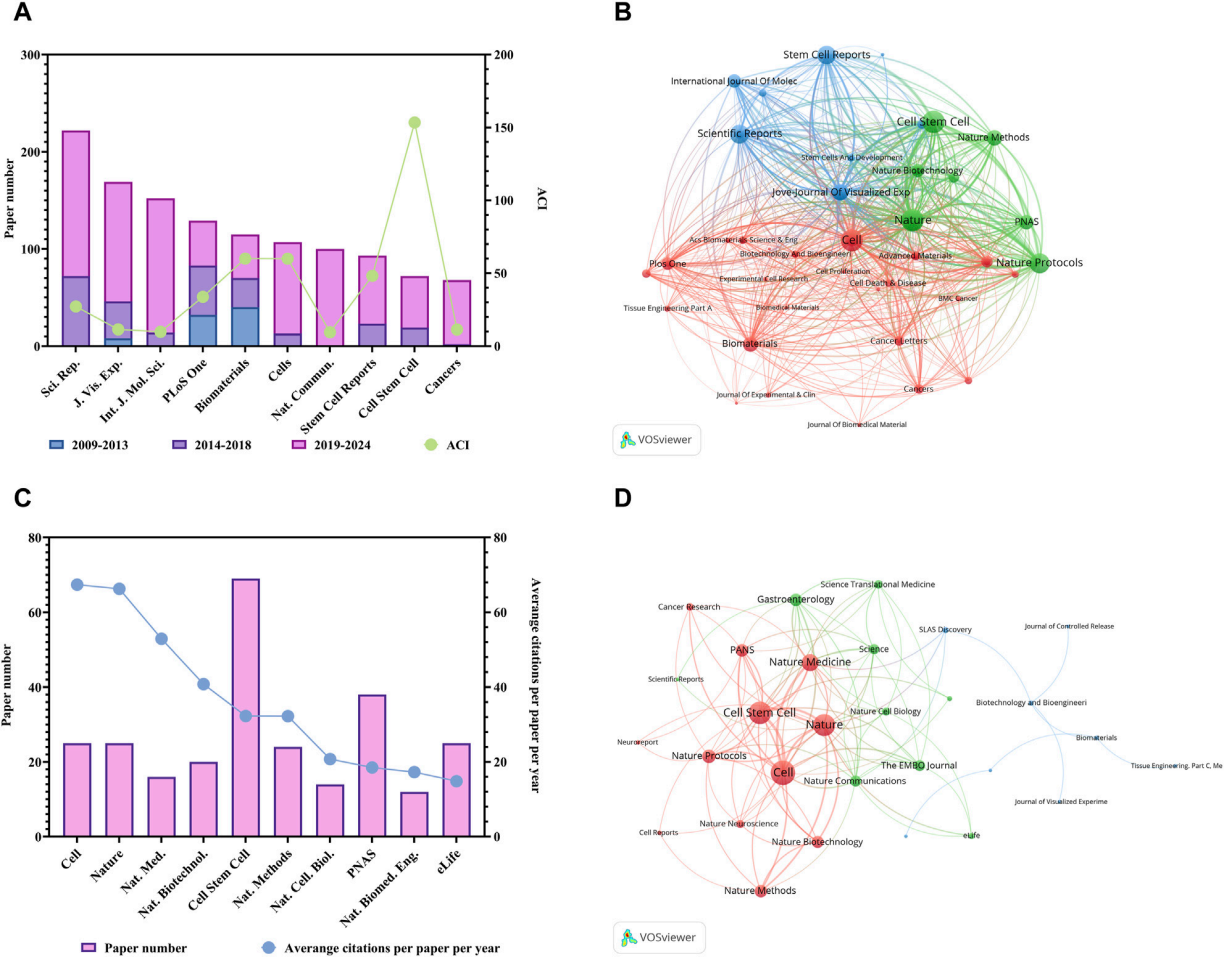
Analysis of journals related to organoids research. **(A)** Paper numbers and ACI of the top 10 productive journals. Scientific Reports, Sci. Rep.; Jove-Journal of Visualized Experiments, J. Vis. Exp.; International Journal of Molecular Sciences, Int. J. Mol. Sci.; Nature Communications, Nat. Commun. **(B)** Co-occurrence network of journals related to organoids by using VOSviewer. The circle size represents the total link strength. The width of the curved line represents the strength of the connection. The journals in the same color are similar research areas. **(C)** Top 10 journals with the most citations per paper per year. Nature Medicine, Nat. Med.; Nature Biotechnology, Nat. Biotechnol.; Nature Methods, Nat. Methods; Nature Cell Biology, Nat. Cell. Biol.; Nature Biomedical Engineering, Nat. Biomed. Eng. **(D)** Co-occurrence network of journals with top papers related to organoids by using VOSviewer. The circle size represents the total link strength. The width of the curved line represents the strength of the connection. The journals in the same color are of similar research areas.

There are a total of 1,206 journals published these 6,222 articles on organoid research. Table 2 listed the top 10 productive journals. Within the group, the journal *Scientific Reports* published the most papers (222 papers, since the year of 2013), then followed by the *Jove-Journal of Visualized Experiments* (169 papers, since the year of 2011) and the *International Journal of Molecular Sciences* (152 papers, since the year of 2012) (Figure 2A). Also, the different proportions of articles published in different historical periods (i.e., 2009–2013; 2014–2018; 2019–2024) were distinguished with different colors in Figure 2A. In addition, *Cell Stem Cell* had the highest ACI (153.46). Furthermore, the co-occurrence analysis of the journals indicated that the three journals including the *Cell stem cell*, *Nature* and *Cell* had the highest TLS (Figure 2B). The top 10 journals with the most citations per paper per year are shown in Figure 2C; Table 3. The journal *Cell* earned the top journal citation per paper per year (67.38), followed by *Nature* (66.28) and *Nature Medicine* (52.92). Moreover, only 25 papers were published in the *Nature*, but the total citation number was as high as 10669. This accounted for 20% of the citations of all papers in the top 10 journals with the most citations per paper per year in organoids (average citation per paper: 426.76; citation per paper per year: 66.28). In addition, *Cell Stem Cell* published the most papers in these 10 highly influential journals (72 papers).

**TABLE 2:** The top 10 productive journals in organoids between 2009 and 2024.

Journals with most papers	Paper numbers	Total citations	ACI	Citations per paper per year	H-index	G-index	If (2023)
Scientific Reports	222	6,033	27.2	6.41	44	65	4.6
Jove-Journal of Visualized Experiments	169	1942	11.5	2.3	21	38	1.2
International Journal of Molecular Sciences	152	1,516	9.9	3.4	19	32	5.6
Plos One	129	4,357	33.8	4.4	38	61	3.7
Biomaterials	115	6,912	60.1	8.3	49	80	14
Nature Communications	107	6,422	60.01	16.7	43	79	16.6
Cells	100	961	9.6	3.9	18	25	6
Stem Cell Reports	93	4,487	48.2	10.6	39	66	5.9
Cell Stem Cell	72	11049	153.46	32.28	49	72	23.9
Cancers	68	775	11.4	3.8	17	24	5.2

**TABLE 3:** The top 10 journals with most citations per paper per year in organoids between 2009 and 2024.

Journals	Paper numbers	Top paper numbers	TPR^a (%)^	Total citations	ACI	Citations per paper per year	If (2023)
Cell	25	12	48	10504	420.16	67.38	64.5
Nature	25	13	52	10669	426.76	66.28	64.8
Nature Medicine	16	8	50	5,039	314.94	52.92	82.9
Nature Biotechnology	20	4	20	3448	172.4	40.81	46.9
Cell Stem Cell	72	13	18.06	11049	153.46	32.28	23.9
Nature Methods	24	4	16.67	4,049	168.71	32.27	48
Nature Cell Biology	16	2	14.29	1760	125.71	20.78	21.3
PNAS	38	5	13.16	4,537	114.66	18.52	11.1
Nature Biomedical Engineering	12	0	0	714	59.5	17.28	28.1
eLife	25	1	4	1824	72.96	14.87	7.7

^a^ Percentage of top papers among all papers in organoids in a journal.

The 100 top papers were listed in Supplementary Table S1 and were published in 36 journals. The citation network of these journals was shown in Figure 2D. The co-occurrence analysis of the journals of top papers showed that the TLS of three journals including the *Nature*, *Cell Stem Cell* and *Cell* were the highest. The *Nature* and *Cell Stem Cell* published the largest number of top papers (13 papers respectively), followed by the *Cell* (12 papers) and the *Nature Medicine* (8 papers). The *Nature* had the highest TPR of 52%, followed by *Nature Medicine* (50%) and *Cell* (48%), which were three journals with TPR exceeding 45% (Table 3). Moreover, there are five other journals with TPR exceeding 10% in the field of organoids, including the *Nature Biotechnology* (20%), *Cell Stem Cell* (18.06%), *Nature Methods* (16.67%), *Nature Cell Biology* (14.29%) and *PNAS* (13.16%), which were considered as major journals on organoids (Table 3). A total of 94 papers published in these journals since 2021 were identified (Supplementary Table S2). Among the major journals, *Cell Stem Cell* published the most papers (34 papers) since 2021.

### 3.3 Countries

**FIGURE 3:**
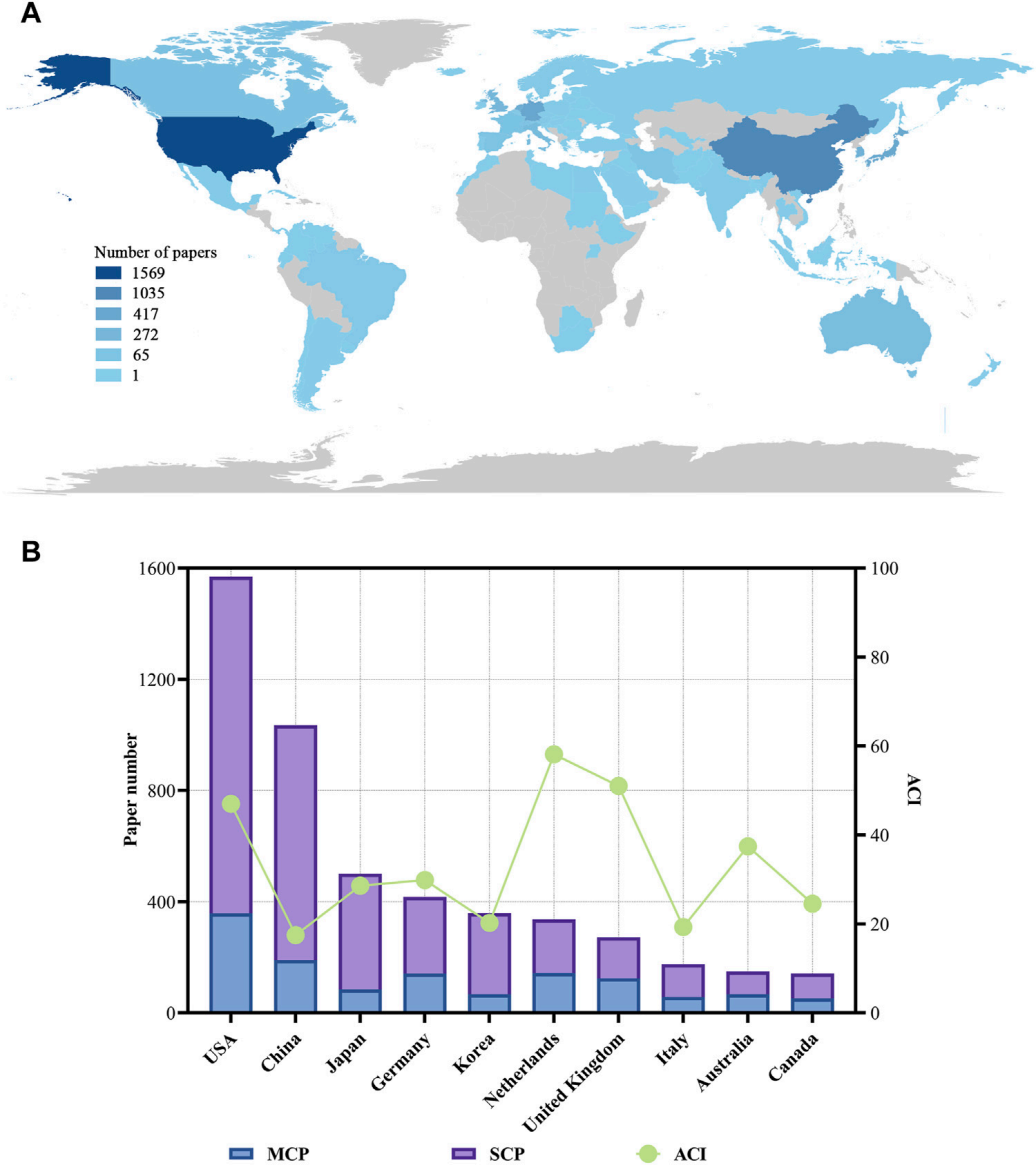
Visualization world map of paper number and total number of papers and ACI in the corresponding author’s countries. **(A)** Visualization world map of paper number. **(B)** Total number of papers and ACI in the corresponding author’s countries. MCP, multiple-country publications; SCP, single-country publications.

**FIGURE 4:**
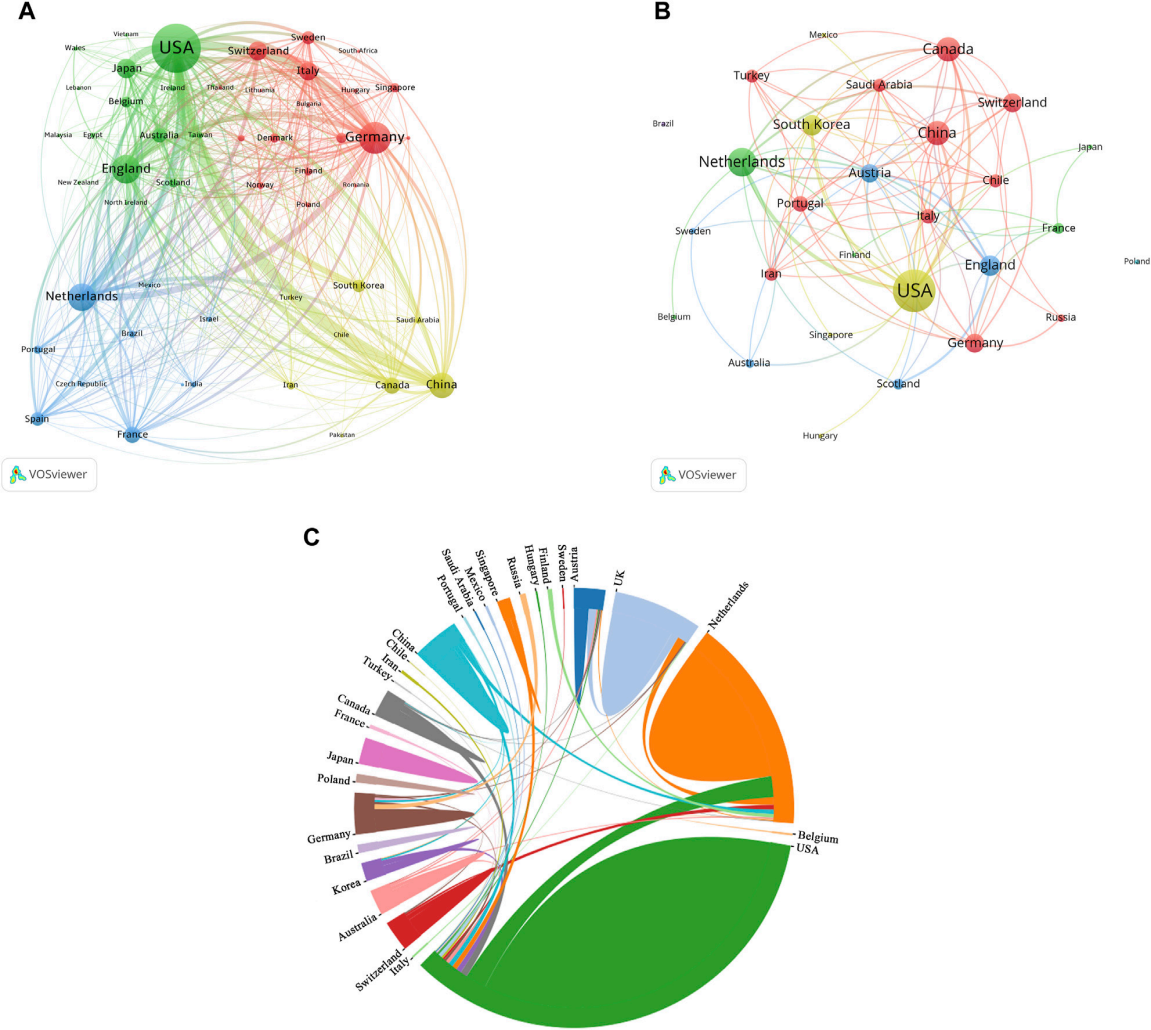
Analysis of countries related to organoids research. **(A)** Co-occurrence network of countries related to organoids by using VOSviewer. The circle size represents the total link strength. The width of the curved line represents the strength of the connection. **(B)** Co-occurrence network of countries with top papers related to organoids by using VOSviewer. The circle size represents the total link strength. The width of the curved line represents the strength of the connection. **(C)** Chordal diagram of the contribution and collaboration of different countries with top papers in the field of organoids.

The authors of these organoids research papers were written by authors from 82 countries or regions, and corresponding authors were from 68 countries or regions (Figure 3A). The top 10 high-output countries or regions were listed in Figure 3B; Table 4. Among them, these corresponding authors from the USA contributed the most papers, accounting for 25.2% of the total (1,569 papers). Followed by China with 1,035 publications (16.6%), and Japan with 500 publications (8.0%). Other notable contributors included Germany, Korea, Netherlands, United Kingdom, Italy, Australia and Canada, each with publication counts ranging from 141 to 417. Moreover, these 10 countries have published 88 top papers, accounting for 88% of the top papers in the field of organoids. In terms of ACI, Netherlands had the highest ACI at 58.17. The United Kingdom (51.02) and the USA (47.03) followed closely behind. Most of the studies were conducted by researchers from a single country, and 26% of the papers were conducted collaboratively. Collaboration in scientific research often manifests as multi-country publications (MCP). The USA had the highest number of MCP at 358 (Figure 3B). Figure 4A illustrated the network visualization of collaboration between countries/regions. The USA was at the center of its cooperation with other countries, most closely with Germany, United Kingdom, Netherlands and China. Although Western developed countries are at the center of the network as research pioneers, while most of the research contributed by Asian countries is relatively new but developing vigorously.

**TABLE 4:** The top 10 productive countries of corresponding authors of papers in organoids between 2009 and 2024.

Country	Articles	Percentage (N/6,222) (%)	Total citations	Multiple-country rate (%)	ACI	Top papers count	Multiple-country top paper rate
USA	1,569	25.20	73786	22.8	47.03	40	32.5%
China	1,035	16.60	18126	18.4	17.51	5	60.0%
Japan	500	8	14299	16.8	28.59	5	20%
Germany	417	6.70	12458	33.8	29.88	5	80%
Korea	359	5.80	7,277	18.7	20.27	3	100%
Netherlands	336	5.40	19544	42.6	58.17	16	50%
United Kingdom	272	4.40	13877	45.6	51.02	10	50%
Italy	175	2.80	3380	32.6	19.31	0	—
Australia	149	2.40	5,582	45	37.46	3	66.7%
Canada	141	2.30	3465	36.9	24.57	1	100%

The authors of the top 100 papers come from 27 countries. Network visualization maps of the collaborative relationships between countries was shown in Figure 4B, with the corresponding authors coming from only 15 countries. Most of the authors of these papers (40) are from the USA (Table 4), indicating that the USA was at the forefront of top papers in the field of organoids, followed by the Netherlands (16 papers) and the United Kingdom (10 papers). In the publication of top papers, the cooperation between the USA and the Netherlands was particularly close (Figure 4C). Despite the high output of papers from China, Korea, Italy, and Canada, the number of top papers published in these countries is relatively low.

### 3.4 Institutions

**FIGURE 5:**
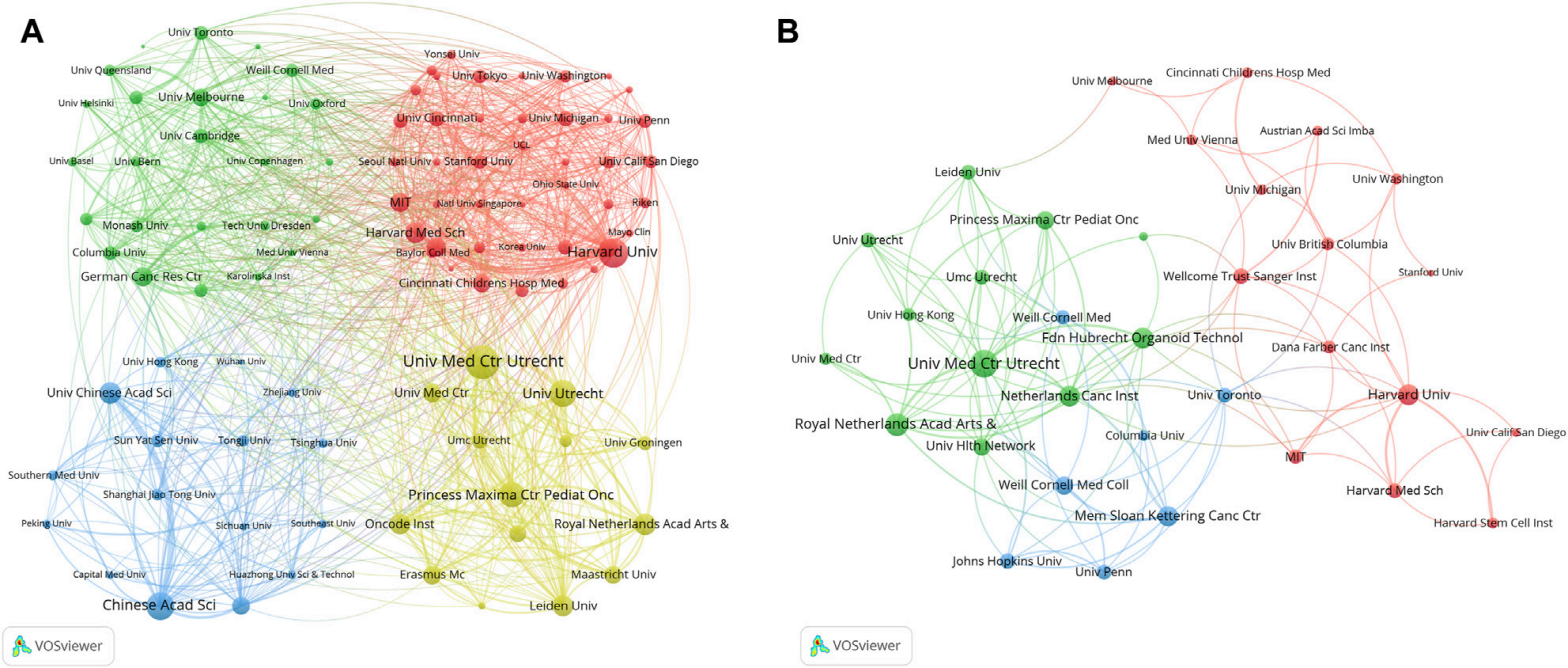
Analysis of institutions related to organoids research by using VOSviewer. The circle size represents total link strength. The width of the curved line represents the strength of the connection. **(A)** Network visualization of institutions related to organoids. The institutions in the same color have stronger collaboration with each other. **(B)** Network visualization of institutions with top papers related to organoids. The institutions in the 1,225 same color have stronger collaboration with each other.

Table 5 listed the top 10 institutions in the field of organoids, with five from the USA, four from the Netherlands, and the rest from China. The University of California System stood out with the most publications (246), followed by Harvard University (227) and Utrecht University (207). The institutional co-occurrence analysis was showed in Figure 5A. Utrecht University Medical Center, Harvard University, and Utrecht University emerged as central nodes. Furthermore, top papers were published by authors from 326 institutions, Utrecht University Medical Center, Royal Netherlands Academy of Arts and Sciences and Harvard University were central nodes (Figure 5B).

**TABLE 5:** The top 10 productive institutions in organoids between 2009 and 2024.

Affiliation	Country	Articles	Percentage (N/6,222 (%)	Top paper numbers
University of California System	USA	246	3.95	9
Harvard University	USA	227	3.65	15
Utrecht University	Netherlands	207	3.33	22
Utrecht University Medical Center	Netherlands	189	3.04	22
Chinese Academy of Sciences	China	162	2.60	22
Helmholtz Association	USA	151	2.43	0
Royal Netherlands Academy of Arts & Sciences	Netherlands	148	2.38	25
Harvard Medical School	USA	144	2.31	8
Hubrecht Institute	Netherlands	143	2.30	24
University System of Ohio	USA	124	1.99	0

### 3.5 Authors

**FIGURE 6:**
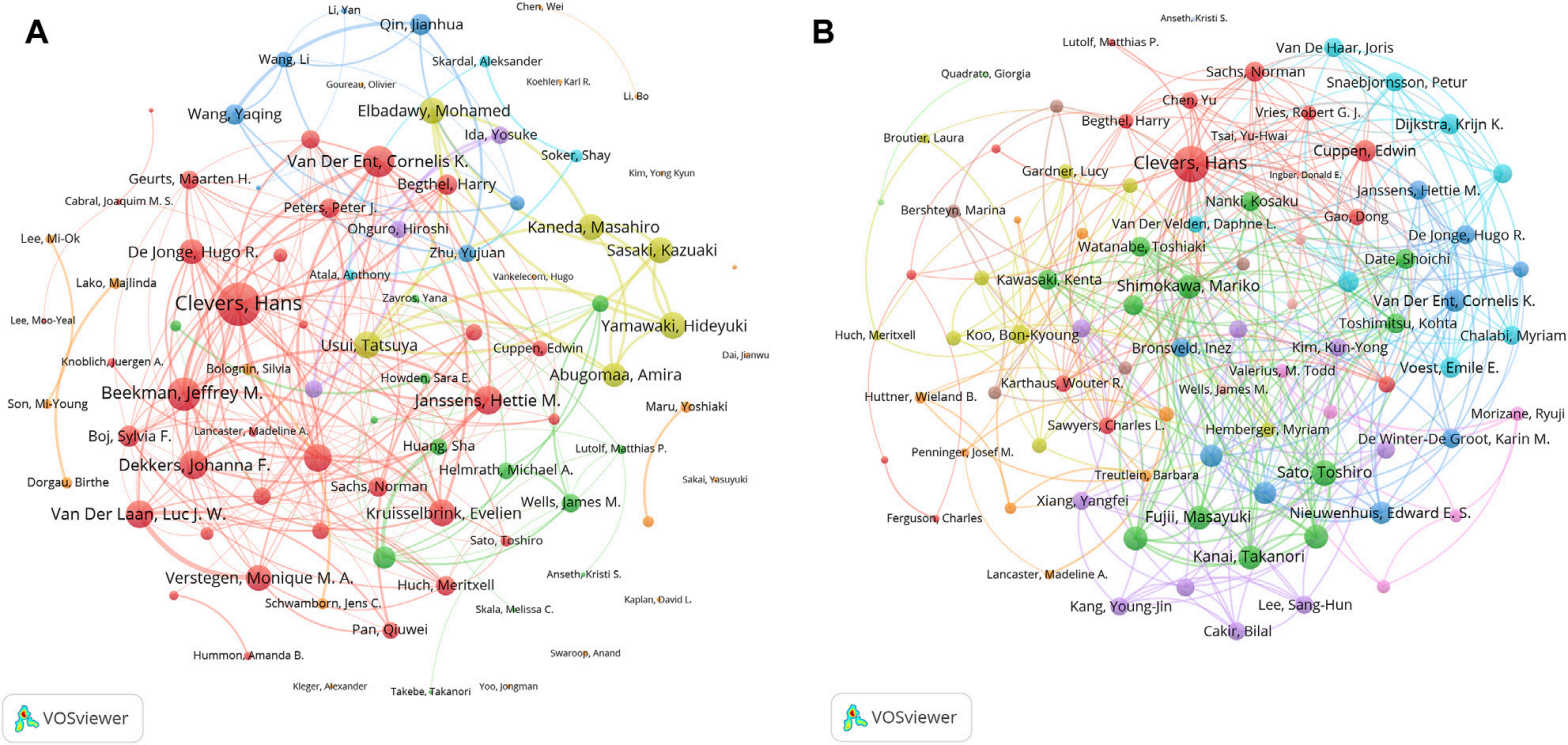
Analysis of authors related to organoids research by using VOSviewer. The circle size represents total link strength. The width of the curved line represents the strength of the connection. **(A)** Network visualization of authors related to organoids. The authors in the same color have stronger collaboration with each other. **(B)** Network visualization of authors with top papers related to organoids. The authors in the same color have stronger collaboration with each other.

A total of 34521 authors participated in the research of organoids. Table 6 listed the top ten productive authors who published at least 30 papers in the field of organoids. The most published author in this field was Clevers, H from Utrecht University (128 papers), who was also the author holding the highest H-index (67) and the most top papers (23), with an average number of citations per paper and local citations of 178.4 and 4,830. In addition, Beekman, JM and Spence, JR published four and three top papers respectively, which also demonstrated their influence in the field of organoids. The author co-occurrence network in Figure 6A showed that Clevers, H and Beekman, JM had the highest TLS, and the analysis of the author co-occurrence network of authors of top papers (Figure 6B) showed that Clevers, H was also in the most critical position.

**TABLE 6:** Authors of at least 30 papers in organoids between 2009 and 2024.

Name	Paper numbers	Total citations	H-index	ACI	Articales fractionalized^a^	Local citations^b^	Top paper numbers
Clevers, H	128	22830	67	178.4	12.57	4,830	23
Kim, J	74	3041	27	41.1	8.64	533	1
Li, Y	52	1,426	20	114.1	5.94	186	0
Wang, J	44	1,211	18	81.9	4.76	175	0
Zhang, Y	41	826	16	27.4	6.15	103	0
Spence, JR	40	3274	21	186.2	4.21	643	3
Lee J	38	1,229	16	72.4	5.69	125	1
Wang, L	38	1,145	15	27.5	3.98	165	0
Wang, Y	38	530	12	175.8	4.64	326	1
Kim, S	37	1876	20	15.9	5.00	142	2
Kim JH	35	585	14	16.7	3.89	70	0
van der Laan, LJW	35	1739	17	49.7	3.27	379	1
Beekman, JM	34	3878	21	114.1	3.00	707	4
Chen Y	34	2,487	15	73.1	4.40	413	3
Liu Y	34	749	15	22.1	3.80	91	0

^a^ Articles Fractionalized = paper number/total number of authors of the papers.

^b^ Citation number in the current dataset (papers in organoids between 2009 and 2024).

### 3.6 Co-cited references analysis

**FIGURE 7:**
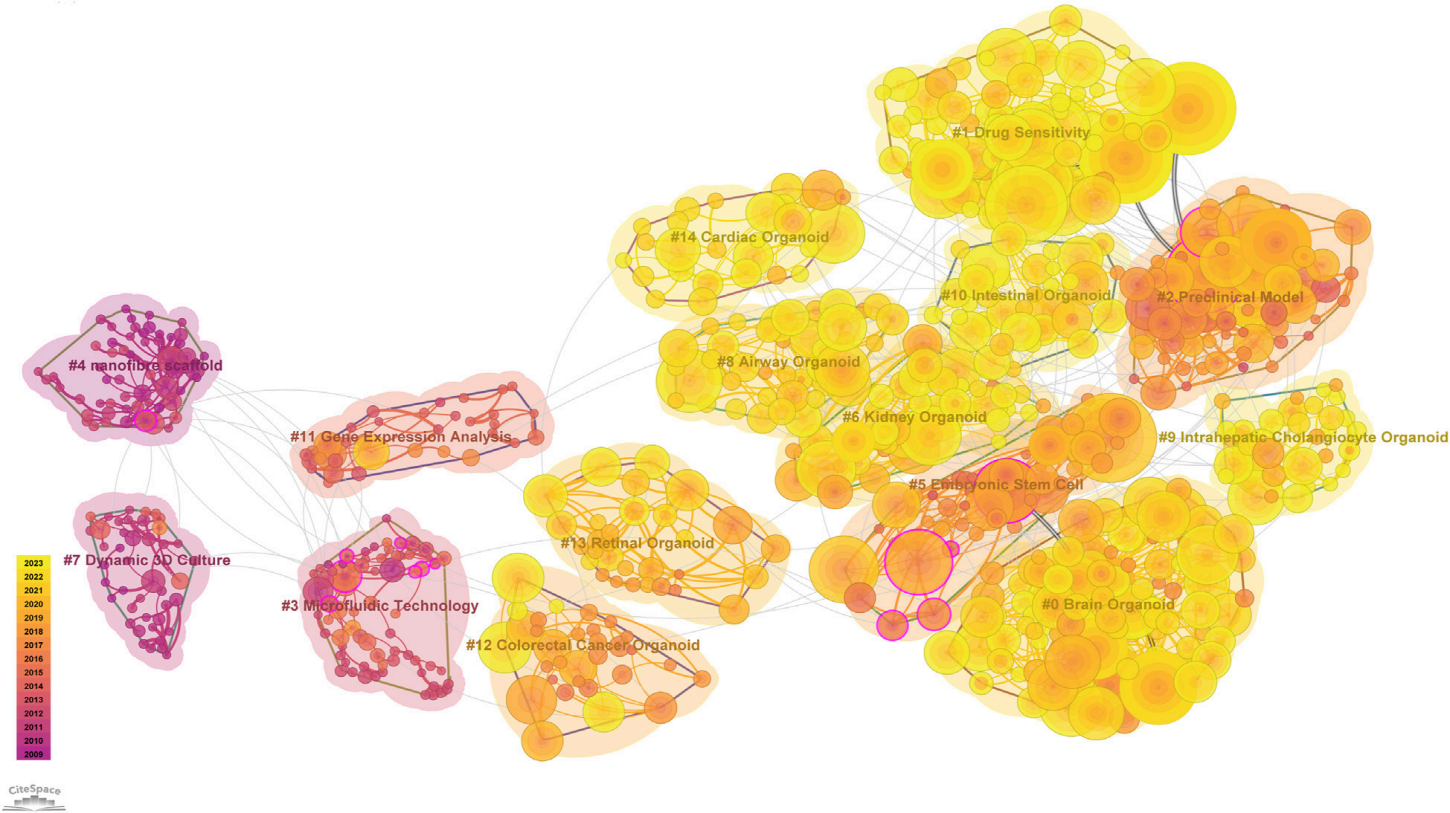
CiteSpace reference co-citation analysis network (Cluster View) in organoids research from 2009 to 2023. The network has a modularity of 0.7943 and an average silhouette score of 0.9387. The labels of each cluster are exhibited beside the blocks. The color of the clusters shows when the co-citation links happened. The purple color means the citing year is relatively early, and the yellow color indicates that the citation time is relatively recent.

We generated a visual representation of the field of organoid research using Latent Semantic Indexing (LSI) technology via CiteSpace and clustering techniques based on title words. As shown in Figure 7, the co-cited references cluster revealed that the field of brain organoids has the highest number of highly cited articles and the highest frequency of citations, followed by the intestinal, hepatic, pulmonary, and renal organoids. In addition, the attention to cardiac, retinal and colorectal cancer organoids was also relatively high. Moreover, recent research has focused on drug sensitivity analysis and preclinical modeling. It is worth noting that although microfluidic technology was first applied in the field of organoids earlier, it has only been widely studied in recent years with the progress of organoid culture technology.

### 3.7 Keywords and research trends

**FIGURE 8:**
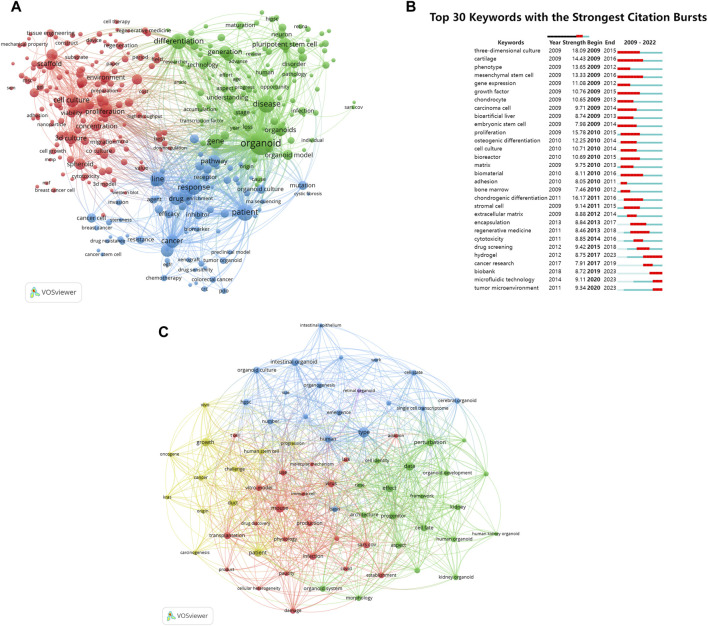
Analysis of keywords and research trends related to organoids research. **(A)** Co-occurrence network of keywords that occurred at least 50 times in the papers by using VOSviewer. The circle size represents the total link strength. The width of the curved line represents the strength of the connection. The keywords in the same color are similar areas. **(B)** Top 30 keywords with the strongest citation bursts in papers of organoids research. The green line indicates the timeline. The intervals in which bursts were found are indicated by red sections on the timeline, indicating the start year, end year, and burst duration according to the average year of publication. **(C)** Co-occurrence network of keywords published in major journals between 2021 and 2024 based on cluster analysis by using VOSviewer. The circle size represents the total link strength. The width of the curved line represents the link strength. The distance between two keywords approximates the relatedness of the nodes.

Utilizing VOSviewer software, we extracted keywords of organoids of the co-occurrence network map for analysis (Figure 8A). These keywords were categorized into three main clusters: “organoids culture” (represented by red nodes), “research on the treatment and mechanism of organoids in cancer” (blue nodes), “organoids research progress” (green nodes). Within the “organoids culture” cluster, the standout keywords were “3D culture”, “scaffold”, and “proliferation”. In the “research on the treatment and mechanism of organoids in cancer” cluster, the studies frequently focused were “patient”, “cancer”, “response”, and “pathway”. In the “organoids research progress” cluster, the representative words were “organoid”, “understanding”, “stage”, and “technology”.

Burst words were used to track the evolution of research topics over time. Figure 8B showed that the previous research mainly focused on exploring and improving the cultivation methods of organoids. In recent years, with the continuous improvement of organoid culture technology, the research hotspots of organoids have gradually shifted to clinical treatment, drug screening, and the application of materials and technologies such as “hydrogel” and “microfluidic technology” in organoids.

An analysis was conducted on 94 organoid papers published in major journals since 2021 by constructing a keyword co-occurrence network (Figure 8C). The keywords that received wider attention than the previous analysis included “patient”, “covid”, and “cancer”.

## 4 Discussion

### 4.1 The development history of organoid technology

The origin of the organoids could be traced back to 1907, when Wilson et al. discovered that the isolated sponge cells could self-organize and regenerate whole organisms (Wilson, 1907). In subsequent chicken embryo experiments, researchers attempted to generate organs from different cell types in the kidneys, skin, and liver (Weiss and Taylor, 1960). In 1981, PSCs were first extracted and established from mouse embryos, which greatly influenced the research progress in the field of organoids (Evans, 1981). Lindberg et al. (Lindberg et al., 1993) and Pellegrini et al. (Pellegrini et al., 1997) subsequently cultured corneal limbal stem cells on 3T3 trophoblast cells and transplanted them into damaged eyes, marking the beginning of the development of 3D organoid technology. In 2009, the landmark study from Clevers, H et al. showed that a Lgr5 positive adult stem cells isolated from mouse intestinal tissue could form a self-renewing culture and differentiate into crypt-villus structures, suggesting that stem cells could self-organize and develop into functional organs under certain conditions (Sato et al., 2009). This was the first report on establishing 3D organoid cultures from a single adult stem cell, laying the foundation for many subsequent organoid works in other systems. Two years later in 2011, it was also the first time that the Clevers, H team successfully established the colon cancer organoids, which was the first research of organoids related to cancer disease (Merlos-Suarez et al., 2011). Other teams have subsequently explored the cultivation system of taste buds, esophagus, fallopian tubes, salivary glands, endometrium and other organoids. In 2020, Lee, J and colleagues successfully cultivated cardiac organoids that could autonomously beat *in vitro.* This type of organoid could spontaneously form a cavity, jump autonomously, without the need for stent support, and could autonomously mobilize cardiac fibroblasts to migrate and repair damage after injury (Lee et al., 2020). Another study in 2021 reported that induced pluripotent stem cell (iPSC) could be used to form brain organoids containing the “optic cups” (which could form the retina) structure. This type of organoids spontaneously developed symmetrical “optic cups” from the anterior part of the brain like region, proving the self-replication ability of iPSCs in highly complex biological processes (Gabriel et al., 2021). Our bibliometric analysis results showed that Clevers, H was the most published author with the highest H-index and the most top papers, who made extraordinary contributions to the field of organoid in organoid technology. The other two influential scientists in the field of organoid research are Beekman, JM and Spence, JR, who have published three and four top papers respectively. Beekman, JM is mainly dedicated to using primary intestinal organoids to study human cystic fibrosis disease. Spence, JR’s research focus is mainly on exploring the developmental process of organs using lung and intestine organoids.

During this time, it is worth noting that, Dr. Valeri, N from the London Cancer Institute in the United Kingdom published a clinical application study in *Science* in 2018, which used tumor organoids cultured *in vitro* for chemotherapy drug sensitivity testing and used it to guide clinical medication, and compared it with the actual efficacy of patients (Vlachogiannis et al., 2018). The results demonstrated that organoids can accurately predict the efficacy of anticancer drugs in patients. Since then, cancer research in the field of organoids has been ongoing, which is also reflected in our analysis of keywords and research trends in the field of organoids. The “research on the treatment and mechanism of organoids in cancer” was one of the main clusters in the keywords analysis of organoids area. Burst words analysis also revealed that “cancer research” has been a hot topic in the field of organoids in recent years. Besides, among the top 10 most cited papers on organoids in our study, 60% were related to cancer. In addition, the frequency of the word “cancer” is relatively high.

### 4.2 The applications of organoids in fundamental biological research area

According to the analysis results of the coupling of the number of organoid related journals and the citation of articles, a considerable amount of research in the top journals focuses on “organoids culture”, which is the cornerstone of other research. Before organoids emerged as one of the models for fundamental research in life medicine, 2D-cell line was one of the most used models. For example, in tumor research, although some mature tumor cell lines can be used as the study of the mechanism and treatment reaction, due to only rare cloning can infinite proliferation and keep the original characteristics of molecular biology, cell lines may undergo substantial genetic changes during batch proliferation, no longer reproducing the genetic heterogeneity of the primary tumor (Caponigro and Sellers, 2011). Besides cell models, animal models are time-consuming to develop and do not faithfully reproduce the disease process in human. As a result, the cell lines and animal model experiments results do not always benefit people.

Organoid culture technology has become a promising method for bridging the gap between 2D cell cultures and animal models. Organoids can be amplified for long-term cultivation, and they are histologically and genetically similar to primitive tissues and can be cryopreserved. Besides, organoids can be cultured from a very small amount of tissue and are easy to perform genetic manipulation (Schutgens and Clevers, 2020; Hendriks et al., 2021). Several studies have explored to model the multi-step tumorigenesis of human intestinal organoids through CRISPR/Cas9 gene editing (Drost et al., 2015; Matano et al., 2015; Ringel et al., 2020). The establishment of organoid models benefits from the advancement of organoid culture technology. The physical characteristics of the cultural environment are crucial for the cultivation of organoids. Only with the appropriate extracellular matrix (ECM) can pluripotent progenitor cells achieve growth and self-organization to form organ-like structures. Therefore, the progress of organoid research benefits from the understanding and study of the biological component of ECM which affects the basic function of cells such as cell growth, proliferation, migration, differentiation and other processes (Pratt, 2021). For example, Bissel and colleagues demonstrated that ECM could regulate the growth and differentiation of mammary epithelial cells. When embedded in ECM hydrogel, mammary epithelial cells could develop tubules and ducts (Roskelley et al., 1994). The cultivation system and conditions vary among different organizations. The research on the components of organoid culture has greatly promoted the research progress of organoids. The organoid culture technology makes it applicable for a wide range of applications in fundamental biological research areas, especially cancer modeling. For example, Li et al. reported the use of epithelial/stromal air liquid interface (ALI) organoids culture methods for multi-step modeling of colon, stomach, and pancreatic tumors (Li et al., 2014).

From the results of bibliometric analysis, the USA contributed the most papers in this field and was at the center of its cooperation with other countries. Notably, Netherlands had the highest ACI at 58.17. In addition, the top 10 institutions in the field of organoids mainly come from developed countries, especially institutions in the USA and the Netherlands including the University of California System, Harvard University, Utrecht University and Utrecht University Medical Center, which play important roles in this field. The efficient, long-term cultivation and cryopreservation of organoids provide possibilities for the research of heterogeneity and evolution of tumors, cancer metastasis, niche factor dependence, and stem cells (Fumagalli et al., 2017). Currently, more and more countries and institutions are participating in the research boom of organoids.

### 4.3 The progress of the most widely studied types of organoids

The co-cited references cluster analysis discovered that the number of highly cited articles and the frequency of citations on brain organoids ranked first, highlighting the important position of neuroscience in the academic community. The high citation count and frequency of articles related to the intestine, liver, lung, and kidney organoids closely followed, reflecting the research activity in the fields of the digestive system, metabolic diseases, respiratory system, and urinary system. Other organoids that have been extensively studied included cardiac, retinal, and colorectal cancer organoids.

The brain organoid technology was first established by Lancaster, MA and colleagues in 2013, who successfully cultivated brain organoids using human PSCs. So far, researchers have successfully established various brain organoid systems, including those simulating the cerebral cortex, midbrain, cerebellum, ventral telencephalon, thalamus, hypothalamus, striatum and hippocampus, bringing great hope for the development of research and treatment in the field of neurological diseases (Chen et al., 2024). In the past decade, intestinal organoid technology has been widely applied in the reproduction of tissue or organ morphology during *in vitro* intestinal physiological processes and the study of the pathogenesis of various intestinal diseases. As a disease model, intestinal organoids played a role in screening diagnostic biomarkers, identifying therapeutic targets, and exploring the epigenetic mechanisms of diseases (Xiang et al., 2024). Moreover, intestinal organoids such as colorectal cancer organoids were widely used for studying cancer genetics, cancer processes, and anti-tumor drug activity (Christin and Shen, 2022). Liver organoids have become valuable tools in various liver disease therapeutic fields. It was reported that liver-derived organoids derived from iPSC-derived bile duct cells could excrete bile acids and exhibit functional secretion, similar to diseases such as Alagille syndrome (Rowe and Daley, 2019). In addition, iPSC-derived liver organoids from specific disease patients could simulate various genetic metabolic disorders and bile duct diseases, which helped to validate *in vitro* drugs for diseases such as polycystic liver disease and cystic fibrosis bile duct disease (Fiorotto et al., 2019). Lung organoids, also referred to as airway organoids or pulmonary organoids, had undergone extensive basic research. Experiments had shown that iPSC-derived lung organoids had structural features similar to native lungs, and their cultivation process was consistent with the characteristics of fetal lung development (Dye et al., 2015). Hence, the emergence of lung organoids had promoted the modeling and research of various diseases such as pulmonary fibrosis, congenital diseases, and neonatal respiratory distress syndrome. In early studies, kidney organoids were generated by PSCs, and directed differentiation methods were developed by following *in vivo* organogenesis to generate kidney organoids from PSCs (Morizane et al., 2015; Takasato et al., 2015). Kidney organoids contained multiple cell types, forming the nephron structure that could be effectively used for research on kidney development, genetic diseases, and infections (Tran et al., 2022). In the past decade, scientists have successfully used tissue engineering techniques to promote the formation of the heart cavity and simulate the complexity of organs (Zhao et al., 2021). The recent significant progress in the field of cardiac organoids was mainly reflected in their ability to simulate *in vivo* cardiac development through self-organization in both spatial and temporal ways, becoming powerful tools for studying cardiac development (Lee et al., 2020; Hofbauer et al., 2021). Retinal organoids have been widely used as tools to simulate eye diseases including retinitis pigmentosa (RP), age-related macular degeneration (AMD) and Leber congenital amaurosis (LCA). Some studies indicated that retinal organoids with eye cup-like structures might contribute to understanding the development and regeneration processes (Quinn et al., 2019). In addition, retinal organoids carrying pathogenic mutations in eye diseases might simulate disease progression *in vitro* and promote the development of effective treatment methods (Martinelli et al., 2022).

### 4.4 The applications of organoids in preclinical research and drug development

Since organoid culture systems have unique advantages in preserving parental gene expression and mutation characteristics, as well as long-term maintenance of parental cell function and biological characteristics *in vitro,* organoid technology provides new opportunities for large-scale drug screening and drug discovery. In our study, the analysis of burst words also indicated that the research hotspots of organoids mainly focused on clinical treatment and drug screening in recent years.

Patient-derived organoids (PDOs) have emerged as an effective *in vitro* model system to facilitate clinical decision-making. For a long time, intratumoral heterogeneity has played a crucial role in cancer progression and treatment resistance (McGranahan and Swanton, 2017), but it has not been maintained at the cellular level. However, organoids can retain their genetic heterogeneity (Boj et al., 2015; Fujii et al., 2016). This characteristic of organoids provides the possibility of being used as a model for drug screening. Organoids in colon cancers (Lemme-Dumit, 2020), pancreatic cancers (Tiriac et al., 2018) gastric cancers (Tiriac et al., 2018), prostate cancer (Gao et al., 2014; Drost et al., 2016) and so on have been established, and the establishment of biobanks for various types of cancer organoids provides potential for drug screening. In 2018, Valeri, N et al. reported that organoids could accurately predict the efficacy of various chemotherapy drugs and targeted drugs in patients with metastatic digestive system tumors (Vlachogiannis et al., 2018), with significantly high specificity (93%) and sensitivity (100%), which marked the beginning of drug screening for organoids. Subsequently, another study established a biobank of organoids derived from locally advanced rectal cancer patients and found that organoids could predict the response of patients to neoadjuvant chemotherapy *in vitro* with an accuracy of 86%, allowing clinicians to tailor treatment plans based on individual patients. Moreover, a research conducted by Lyudmyla revealed that the drug sensitivity of organoids derived from pancreatic ductal adenocarcinoma (PDAC) patients was highly similar to the clinical response of patients, and the drug response predicted by PDO was also related to tumor cellularity (Demyan et al., 2022). It is worth noting that organoids have become a powerful force in high-throughput drug screening (Lampart et al., 2023), which accelerated the development of the clinical transformation of organoid technology. Driehuis et al. created a biobank of pancreatic cancer organoids that retained the histological characteristics of primary tumors and carried the common genetic changes in original tumors. Using this biobank, 76 compounds were selected for high-throughput drug screening, and a series of effective targeted therapies for PDO were determined (Driehuis et al., 2019). Moreover, Zhang et al. developed a high-throughput screening platform based on human liver organoid-on-a-chip for drug-induced liver injury, which integrated multiple omics including biomarker/analyte detection, high-content imaging-enabled phenotyping, and single-cell RNA sequencing (Zhang et al., 2023).

Organoids also play important roles in accelerating drug development. In the early stages of drug discovery, organoids could provide powerful predictive preclinical models for drugs that are effective for most patients and even tumors with specific mutations (Herpers et al., 2022). In addition, the organoids of healthy tissues could be used to detect the side effects of drugs on organs (Shinozawa et al., 2021). The hepatotoxicity of drugs is one of the main reasons for the failure of drug development, and liver organoids could be used to test the hepatotoxicity of potential new drugs (Harrison et al., 2021). A recent study explored the hepatitis E virus (HEV)-host interactions in liver organoids and discovered that brequinar and homoharringtonine are potent anti-HEV drugs (Li P. et al., 2022). A locally applied antibiotic, namely, bacitracin, has been discovered that could inhibit the activity of *Clostridium difficile* and its toxin B in intestinal organoids infected with bacteria (Zhu et al., 2019). Moreover, Organoids can simulate genetic disease models and be used to develop new drugs for treating these diseases. Cystic fibrosis (CF) is a monogenic hereditary disease characterized by functional impairments in the lungs, pancreas, liver, intestines, and reproductive system. Recently, Organoids derived from CF patients were used for high-throughput compound determination to determine potential drugs for treating individuals with rare CF transmembrane conductance regulator mutations (Spelier et al., 2023). The establishment of other genetic organoid models, such as autosomal dominant polycystic kidney disease, hereditary retinal disease, and primary microcephaly, has brought dawn to the etiology, pathogenesis, and development of therapeutic drugs for genetic diseases (Dhaliwal et al., 2021; Lewis-Israeli et al., 2021; Li S. R. et al., 2022). Besides, organoids also play significant roles in the study of explosive epidemics. It is worth noting that in the process of fighting against COVID-19 in the past few years, organoids have contributed to host-pathogen interactions, to the study of the cellular tropism of the virus in different organs and identified potential drug candidates that impact the disease (Kim et al., 2022). In our study, an analysis was conducted on papers of organoids published in major journals since 2021 suggesting that the keyword “covid” received wider attention. Notably, the USFDA has approved organoids for clinical trials in place of patients for the first time. In February 2022, the USFDA officially approved the cold agglutinin syndrome drug Enjaymo for the treatment of autoimmune demyelinating disease based on preclinical efficacy data of human organoids (Yu et al., 2022), suggesting that the use of organoids could accelerate the conversion of the best drugs (Rumsey et al., 2022). As Professor Thomas Hartung says: “This illustrates that the whole field of bioengineering human organs can now be described as one of the examples of an ongoing scientific revolution”.

### 4.5 The application of materials and technologies in organoids

The development of organoids and materials science is complementary. The analysis of burst words shown in Figure 8B suggested that the keywords “hydrogel” and “microfluidic technology” appeared more frequently in recent years. The traditional materials used in organoid cultures were static, which may lead to the accumulation of biochemical waste in the central part of organoids and cannot reproduce the cellular microenvironment well. With the rapid development of biomaterials, functional biomaterials represented by hydrogels provide new opportunities in the field of organoid culture and microenvironment reproduction. Hydrogel is a polymer with high water content. Its composition is relatively clear, most of which have adjustable physical and chemical properties and good biocompatibility. It can be used as a bionic 3D ECM to simulate the complex cellular tissue microenvironment and guide a variety of cell behaviors such as cell adhesion, migration, proliferation and differentiation.

Recently, microfluidic organoid-on-a-chip models have been developed for modeling various organs *in vitro*, including the lungs (Shrestha et al., 2020), liver (Jang et al., 2019), kidneys (Wilmer et al., 2016), and heart (Abulaiti et al., 2020). The organoid-on-a-chip models based on design and engineering principles can accurately control the microenvironment and simulate the organoid-level functions *in vitro* (Bhatia and Ingber, 2014). In addition, different microfluidic chips can be interconnected to construct on-chip body models that can simulate multi-organ interactions and related key physiological, biophysical, and biochemical cues (Park et al., 2019; Monteiro et al., 2022). So far, several microfluidic chips have been developed for culturing organoids to study applications, such as drug efficacy testing, drug penetration into tumors, and angiogenesis (Huang et al., 2017; Lim and Park, 2018; Chou et al., 2020). Moreover, it is worth noting that the integration of microfluidic technology and 3D bioprinting technology is becoming a promising revolutionary way to simulate the complex cellular microenvironment including tumor microenvironment (TME). 3D bioprinting technology can quickly, automatically, and repeatably control the complex manufacture of anatomical-sized tumor models. These structures are constructed from well-designed tumor mimetic bioinks, combining tumor/stromal cells with various ECM mimetic biomaterials (Radhakrishnan et al., 2020). The use of 3D bioprinting to construct complex tissues and the introduction of dynamic environments through microfluidic systems are more conducive to understanding the pathophysiology of human tumors and screening more effective treatment methods, surpassing the limitations of currently available static 3D tumor models (Monteiro et al., 2022; Miserocchi et al., 2023). For example, 3D bioprinting technology could engineer the vascular like tubular structures for assembling 3D vascular competent models on the fly during biofabrication, which promoted the personalization of vascularized microfluidic channels in real-time (Cao et al., 2019; Grigoryan et al., 2019). In addition, 3D bioprinting could also include ECM-mimetic hydrogel biomaterials, and spatially controlled deposition of cell-laden ECM-mimetic hydrogel bioinks within a microfluidic device, eliminating the variability of traditional cell seeding methods in the microfluidic chip (Monteiro et al., 2022).

### 4.6 The application of organoid-immune co-culture models in precision medicine

The co-cultivation of immune cells and organoids, especially tumor organoids, deepens our understanding of tumor immunology and may pave the way for more effective personalized healthcare. However, this method is still in the early stages of development and the research papers may not seem sufficient to be recognized by bibliometric methods, but we believe it still needs to be mentioned. In the past decade, treatment for the immune system has brought new hope to the field of cancer treatment. Immune checkpoint inhibitors and other therapies have been approved as first-line treatment drugs for various solid tumors. However, due to tumor heterogeneity and treatment resistance, the overall clinical efficacy of most immunotherapies is limited (Magre et al., 2023). Therefore, predicting patient-specific reactions is of great value for the effective use of expensive immunotherapy drugs. As mentioned above, microfluidic culture technology can simulate the tumor immune microenvironment *in vitro* systems, effectively solving the problems of traditional organoid models lacking TME including immune cells and stromal cells. At present, co-culture techniques combining tumor organoids and immune cells including cytotoxic T lymphocytes and dendritic cells, NK cells, macrophages, and lymphocytes have been developed (Yuan et al., 2023), providing multiple pathways for studying individualized drug responsiveness. For example, Tsai et al. co-cultured PDAC organoids with cancer-associated fibroblasts and CD3^+^ T lymphocytes to develop a specific TME for PDAC (Tsai et al., 2018). Another study reported the addition of peripheral blood lymphocytes and co-culture with non-small cell lung cancer organoids, providing a clinically feasible method for producing patient-specific T cells for adoptive T cell transfer (Dijkstra et al., 2018). Recently, a new co-culture strategy has been developed, which used tumor antigens to stimulate antigen-presenting dendritic cells (DCs), then co-culture with CD8^+^ T cells to promote cell lysis and proliferation of these T cells, followed by co-culture with gastric cancer organoids from patient sources. This approach effectively predicts the efficacy of precision medicine to achieve better prognosis for gastric cancer patients (Zavros et al., 2021). Moreover, co-cultivation of PDO with immune cells combined with checkpoint blockade inhibitors has been applied in multiple cancer precision medicine studies, providing significant insights for predicting the precision treatment effect of PDO (Chakrabarti et al., 2021a; Chakrabarti et al., 2021b; Meng et al., 2021). Thus it can be seen that organoid-immune co-cultures may deepen our understanding of tumor immunology and pave the way for more effective personalized healthcare.

### 4.7 The pitfalls, challenges and prospects of organoid technology

As mentioned above, organoids have successfully addressed the limitations of 2D cell culture and animal models. Human-derived organoids have the ability to faithfully replicate complex pathological and physiological processes within the body. This ability has broad application prospects in organ development, disease modeling, precision medicine, and drug discovery. Although significant progress has been made in the field of organoids over the past decade, it still faces pitfalls and challenges.

The ethical issues of organoids are the pitfalls that cannot be ignored. On the one hand, researchers can only conduct research on organoids with the permission of the donor. Researchers should follow ethical standards and legal regulations to ensure the ethical compliance of their research. This includes obtaining informed consent from patients, respecting the rights of donors and research participants, and protecting their privacy and personal data (Boers et al., 2018; Lensink et al., 2021). On the other hand, the ethical challenge of commercializing organoids is also an issue that exists. The survey showed that the majority of patients participating in the study hoped to understand the experimental research on organoids, as well as the related profits and drug pricing that might be involved in the future, which could be solved by providing benefits to donors, including financial support or free experience of new therapies based on organoid research (de Jongh et al., 2022). Therefore, ethical issues in organoid research need to be addressed through adherence to ethical guidelines, ethical review, transparent communication, and other means to ensure the ethical compliance of research and the protection of participant rights.

Organoid technology still has a few challenges that hinder its broader application. One of the main components of organoid culture is the use of a matrix that facilitates organoid development. Matrigel and basement membrane extracts have compositional differences between batches, and this batch variability can affect the reproducibility of the results (Phipson et al., 2019). Therefore, optimizing morphological gradients, tissue-specific cell-ECM interactions, and local biochemical and biophysical properties are crucial for minimizing inter-batch heterogeneity to the greatest extent possible (Zhao et al., 2022). For example, using synthetic matrices to achieve more complete control over composition and stiffness, and employing decellularized tissue to create tissue-specific matrices to optimize ECM composition, are effective ways to overcome internal heterogeneity within organoids (Kozlowski et al., 2021; Rezakhani et al., 2021). The current organoid technology is also unable to model multiorgan pathologies. Recent studies have shown that the use of co-culture methods and organoid chip technology provided the possibility to solve this problem (Papp et al., 2024). In addition, although organoids derived from PSCs are better at recapitulating the different cell types and cell interactions of developing organs, they have failed to demonstrate adult tissue structure and function as well as cell maturation. The diffusion of nutrients and oxygenation are two important components in tissue maturation (Tabibzadeh et al., 2023). When organoids grow for a long time, they begin to undergo cell apoptosis or necrosis due to hypoxia (Giandomenico et al., 2019; Qian et al., 2020). To overcome this problem, a new organoid perfusion strategy needs to be designed, such as by designing perfusable open-end structures that overcome the accumulation of dead cells and cell debris in the cystic organoid lumina and wash out cell debris by inducible flow (Zhao et al., 2022).

Looking ahead, the trend in organoid technology is to develop more complex models that faithfully recapitulate *in vivo* structure and function, in terms of the recapitulating cell types over time, tissue architecture, measurable molecular events and phenotypic functions. Furthermore, organoid technology should be integrated with other technologies, such as 3D bioprinting technology and microfluidic technology, which may accelerate the development of organoids and improve their accuracy in reproducing human physiological and pathological conditions.

## 5 Advantages and limitations

Our study is the first to harness bibliometric analysis in exploring the latest research process in the entire field of organoids, distinguishing itself from traditional literature reviews. There are several strengths to our approach. Firstly, our systematic search strategy and rigorous quantitative statistical methodologies offer a more comprehensive and insightful perspective. Secondly, the use of tools such as CiteSpace, VOSviewer, and the R package bibliometrix ensures holistic data extraction, meticulous bibliometric evaluation, and enhanced visualization. Lastly, our multifaceted approach provides a panoramic view of the landscape, emphasizing the need for more in-depth qualitative analysis to provide a rounded understanding of the field.

However, there are limitations to our study. The exclusive reliance on the WoSCC database might have omitted certain pertinent literature, especially those with fewer citations. Additionally, the bibliometric methodologies we employed, while robust, might have overlooked nuanced insights, specific authorial perspectives, and forward-looking opinions embedded within the full texts. There are also potential inconsistencies between the results of our bibliometric analysis and the status of actual studies, given the dynamic nature of the database.

## 6 Conclusion

This study provided a comprehensive and timely bibliometric analysis covering the entire organoid research landscape. Our findings highlight a growing trend in publications, with the USA and Netherlands leading in this field. The University of California System, Harvard University, Utrecht University and Utrecht University Medical Center have emerged as pivotal contributors, with numerous publications and most top paper numbers. Key authors in the field include Clevers, H, Beekman, JM and Spence JR. Through keywords co-occurrence analysis, we identified three clusters: “organoids culture”, “research on the treatment and mechanism of organoids in cancer” and “organoids research progress”. Notably, the research hotspots of organoids have gradually shifted to clinical treatment, drug screening, and the application of materials and technologies such as “hydrogel” and “microfluidic technology” in organoids. In terms of cancer treatment, due to the deepening understanding of the diversity of tumor infiltrating cells and the heterogeneity within tumors, personalized healthcare has become increasingly important. The organoid-immune co-culture model will play an increasingly important role in the field of tumor-immune interactions and may show broad prospects in developing patient specific treatment methods. In summary, organoids provide unprecedented opportunities for studying human development, physiology, and diseases, providing better options for drug screening and personalized drug therapy. The highlight of this article is that we provide an in-depth statement and discussion on the development process and application of organoid technology, based on the bibliometric analysis results of organoid publications. We revealed a comprehensive blueprint of the organoid research area from the initial of the first organoids established from adult stem cells, covering the following aspects: the development history of organoid technology, the progress of the most widely studied types of organoids, the organoid research from bench to bedsides (including organoids as good models for fundamental research, and the advantages of organoids as potential preclinical models in drug screening and development in clinical research), the development of culture materials and microfluidic technologies, the emerging organoid-immune co-cultures trends, and the pitfalls, challenges and prospects of organoid technology, providing readers straightforward and convenient access to the organoid research field.

## Data Availability

The original contributions presented in the study are included in the article/Supplementary Material, further inquiries can be directed to the corresponding authors.
