# Supplementary material for: Interpretation of the past, present, and future of organoid technology: an updated bibliometric analysis from 2009 to 2024
Source: Front Cell Dev Biol. 2024 Aug 13;12:1433111. doi: 10.3389/fcell.2024.1433111 (PMC11347291; doi:10.3389/fcell.2024.1433111)
Supplement: Supplementary file 1 [file Table1.DOCX]

**Supplementary Table 1.** The 100 most cited papers in organoids between 2009 and 2024.

| **Rank** | **Title** | **Corresponding**  **Author** | **Journal** | **Year** | **Total citations** | **Average citations**  **per year (rank)** |
| --- | --- | --- | --- | --- | --- | --- |
| 1 | Cerebral organoids model human brain development and microcephaly | Knoblich, JA | Nature | 2013 | 3035 | 286.77(1) |
| 2 | Long-term Expansion of Epithelial Organoids From Human Colon, Adenoma, Adenocarcinoma, and Barrett's Epithelium | Sato, T | Gastroenterology | 2011 | 2397 | 193.05(2) |
| 3 | Hydrogels as Extracellular Matrix Mimics for 3D Cell Culture | Anseth, KS | Biotechnology and Bioengineering | 2009 | 1959 | 132.81(9) |
| 4 | Prospective Derivation of a Living Organoid Biobank of Colorectal Cancer Patients | Garnett, MJ | Cell | 2015 | 1472 | 165.08(5) |
| 5 | Organoid Models of Human and Mouse Ductal Pancreatic Cancer | Clevers, H | Cell | 2015 | 1363 | 147.35(7) |
| 6 | Brain-Region-Specific Organoids Using Mini-bioreactors for Modeling ZIKV Exposure | Song, HJ; Ming, GL | Cell | 2016 | 1343 | 169.64(4) |
| 7 | Patient-derived organoids model treatment response of metastatic gastrointestinal cancers | Valeri, N | Science | 2018 | 1059 | 171.73(3) |
| 8 | A Living Biobank of Breast Cancer Organoids Captures Disease Heterogeneity | Clevers, H | Cell | 2018 | 1029 | 164.64(6) |
| 9 | Organoid Cultures Derived from Patients with Advanced Prostate Cancer | Chen, Y | Cell | 2014 | 1015 | 105.91(16) |
| 10 | Functional Repair of CFTR by CRISPR/Cas9 in Intestinal Stem Cell Organoids of Cystic Fibrosis Patients | Clevers, H | Cell Stem Cell | 2013 | 957 | 92.61(30) |
| 11 | Kidney organoids from human iPS cells contain multiple lineages and model human nephrogenesis | Takasato, M | Nature | 2015 | 954 | 112.24(14) |
| 12 | Functional cortical neurons and astrocytes from human pluripotent stem cells in 3D culture | Pasca, SP | Nature Methods | 2015 | 912 | 104.23(19) |
| 13 | Generation of cerebral organoids from human pluripotent stem cells | Lancaster, MA | Nature Protocols | 2014 | 911 | 95.89(28) |
| 14 | Zika virus impairs growth in human neurospheres and brain organoids | Garcez, PP; Rehen, SK | Science | 2016 | 824 | 104.08(20) |
| 15 | Human primary liver cancer-derived organoid cultures for disease modeling and drug screening | Huch, M | Nature Medicine | 2017 | 797 | 125.84(11) |
| 16 | A three-dimensional human neural cell culture model of Alzheimer's disease | Kim, DY | Nature | 2014 | 796 | 84.53(35) |
| 17 | Organoid Modeling of the Tumor Immune Microenvironment | Kuo, CJ | Cell | 2018 | 763 | 143.06(8) |
| 18 | 2D and 3D cell cultures - a comparison of different types of cancer cell cultures | Kolenda, T | Archives of Medical Science | 2018 | 758 | 129.94(10) |
| 19 | Modeling colorectal cancer using CRISPR-Cas9-mediated engineering of human intestinal organoids | Sato, T | Nature Medicine | 2015 | 751 | 82.68(40) |
| 20 | Cell diversity and network dynamics in photosensitive human brain organoids | Quadrato, G; Arlotta, P | Nature | 2017 | 725 | 104.82(18) |
| 21 | A functional CFTR assay using primary cystic fibrosis intestinal organoids | Beekman, JM | Nature Medicine | 2013 | 703 | 65.4(56) |
| 22 | An in vivo model of functional and vascularized human brain organoids | Gage, FH | Nature Biotechnology | 2018 | 684 | 115.61(13) |
| 23 | Human cerebral organoids recapitulate gene expression programs of fetal neocortex development | Pääbo, S | PANS | 2015 | 660 | 79.2(43) |
| 24 | Modelling human development and disease in pluripotent stem-cell-derived gastric organoids | Wells, JM | Nature | 2014 | 651 | 69.75(50) |
| 25 | Organoid Profiling Identifies Common Responders to Chemotherapy in Pancreatic Cancer | Tuveson, DA | Cancer Discovery | 2018 | 578 | 103.52(21) |
| 26 | Generation of Tumor-Reactive T Cells by Co-culture of Peripheral Blood Lymphocytes and Tumor Organoids | Voest, EE | Cell | 2018 | 577 | 103.34(22) |
| 27 | Designer matrices for intestinal stem cell and organoid culture | Lutolf, MP | Nature | 2016 | 566 | 76.31(47) |
| 28 | Nephron organoids derived from human pluripotent stem cells model kidney development and injury | Morizane, R | Nature Biotechnology | 2015 | 566 | 67.25(53) |
| 29 | Multisensor-integrated organs-on-chips platform for automated and continual in situ monitoring of organoid behaviors | Zhang, YS; Khademhosseini, A | PANS | 2017 | 543 | 76.66(45) |
| 30 | Three-Dimensional Cell Cultures in Drug Discovery and Development | Eglen, RM | Slas Discovery | 2017 | 523 | 76.54(46) |
| 31 | A Colorectal Tumor Organoid Library Demonstrates Progressive Loss of Niche Factor Requirements during Tumorigenesis | Sato, T | Cell Stem Cell | 2016 | 522 | 66.64(55) |
| 32 | In vitro generation of human pluripotent stem cell derived lung organoids | Spence, JR | Elife | 2015 | 522 | 57.47(69) |
| 33 | Long-term expanding human airway organoids for disease modeling | Clevers, H | Embo Journal | 2019 | 521 | 100.84(25) |
| 34 | Zika Virus Depletes Neural Progenitors in Human Cerebral Organoids through Activation of the Innate Immune Receptor TLR3 | Rana, TM | Cell Stem Cell | 2016 | 520 | 67.83(52) |
| 35 | Identification of Multipotent Luminal Progenitor Cells in Human Prostate Organoid Cultures | Clevers, HC | Cell | 2014 | 514 | 53.63(74) |
| 36 | The Use of Whole Organ Decellularization for the Generation of a Vascularized Liver Organoid | Soker, S | Hepatology | 2011 | 507 | 38.51(86) |
| 37 | Ductal pancreatic cancer modeling and drug screening using human pluripotent stem cell- and patient-derived tumor organoids | Muthuswamy, SK | Nature Medicine | 2015 | 505 | 60(66) |
| 38 | Midbrain-like Organoids from Human Pluripotent Stem Cells Contain Functional Dopaminergic and Neuromelanin-Producing Neurons | Ng, HH | Cell Stem Cell | 2016 | 493 | 64.3(57) |
| 39 | Individual brain organoids reproducibly form cell diversity of the human cerebral cortex | Arlotta, P | Nature | 2019 | 492 | 101.79(23) |
| 40 | Modelling kidney disease with CRISPR-mutant kidney organoids derived from human pluripotent epiblast spheroids | Freedman, BS | Nature Communications | 2015 | 482 | 56.71(70) |
| 41 | Three-dimensional tissue culture based on magnetic cell levitation | Killian, TC | Nature Nanotechnology | 2010 | 480 | 34.29(89) |
| 42 | Guided self-organization and cortical plate formation in human brain organoids | Lancaster, MA; Knoblich, JA | Nature Biotechnology | 2017 | 479 | 70.96(49) |
| 43 | Flow-enhanced vascularization and maturation of kidney organoids in vitro | Lewis, JA | Nature Methods | 2019 | 470 | 92.46(32) |
| 44 | Engineering of human brain organoids with a functional vascular-like system | Park, IH | Nature Methods | 2019 | 464 | 105.06(17) |
| 45 | Tumor Evolution and Drug Response in Patient-Derived Organoid Models of Bladder Cancer | Shen, MM | Cell | 2018 | 464 | 77.33(44) |
| 46 | A Human Pluripotent Stem Cell-based Platform to Study SARS-CoV-2 Tropism and Model Virus Infection in Human Cells and Organoids | Pan, FC; Evans, T; Chen, SB | Cell Stem Cell | 2020 | 459 | 122.4(12) |
| 47 | A Patient-Derived Glioblastoma Organoid Model and Biobank Recapitulates Inter- and Intra-tumoral Heterogeneity | Ming, GL; Song, HJ | Cell | 2020 | 452 | 106.35(15) |
| 48 | Long-Term Expansion of Functional Mouse and Human Hepatocytes as 3D Organoids | Clevers, H | Cell | 2018 | 448 | 82.71(39) |
| 49 | Culture and establishment of self-renewing human and mouse adult liver and pancreas 3D organoids and their genetic manipulation | Huch, M | Nature Protocols | 2016 | 436 | 57.49(68) |
| 50 | Development of a primary human Small Intestine-on-a-Chip using biopsy-derived organoids | Ingber, DE | Scientific Reports | 2018 | 426 | 69.08(51) |
| 51 | An organoid platform for ovarian cancer captures intra- and interpatient heterogeneity | Clevers, H | Nature Medicine | 2019 | 425 | 86.44(34) |
| 52 | Fused cerebral organoids model interactions between brain regions | Knoblich, JA | Nature Methods | 2017 | 421 | 62.37(60) |
| 53 | Human blood vessel organoids as a model of diabetic vasculopathy | Wimmer, RA; Penninger, JM | Nature | 2019 | 417 | 79.43(42) |
| 54 | Self-Organization of Polarized Cerebellar Tissue in 3D Culture of Human Pluripotent Stem Cells | Muguruma, K | Cell Reports | 2015 | 417 | 45.49(83) |
| 55 | A Simple Hanging Drop Cell Culture Protocol for Generation of 3D Spheroids | Foty, R | Jove-Journal of Visualized Experiments | 2011 | 405 | 31.35(91) |
| 56 | Patient-derived organoids can predict response to chemotherapy in metastatic colorectal cancer patients | Voest, EE | Science Translational Medicine | 2019 | 398 | 88.44(33) |
| 57 | Fusion of Regionally Specified hPSC-Derived Organoids Models Human Brain Development and Interneuron Migration | Park, IH | Cell Stem Cell | 2017 | 394 | 59.85(67) |
| 58 | A Comprehensive Human Gastric Cancer Organoid Biobank Captures Tumor Subtype Heterogeneity and Enables Therapeutic Screening | Yang, HHN; Leung, SY | Cell Stem Cell | 2018 | 393 | 73.69(48) |
| 59 | Organoid culture systems for prostate epithelial and cancer tissue | Drost, J; Clevers, H | Nature Protocols | 2016 | 393 | 48.12(77) |
| 60 | Human Pancreatic Tumor Organoids Reveal Loss of Stem Cell Niche Factor Dependence during Disease Progression | Sato, T | Cell Stem Cell | 2018 | 383 | 62.96(58) |
| 61 | Patient-derived lung cancer organoids as in vitro cancer models for therapeutic screening | Jang, SJ | Nature Communications | 2019 | 382 | 83.35(38) |
| 62 | Complex Oscillatory Waves Emerging from Cortical Organoids Model Early Human Brain Network Development | Muotri, AR | Cell Stem Cell | 2019 | 380 | 84.44(36) |
| 63 | Long-term, hormone-responsive organoid cultures of human endometrium in a chemically defined medium | Turco, MY | Nature Cell Biology | 2017 | 379 | 54.8(72) |
| 64 | Infection of bat and human intestinal organoids by SARS-CoV-2 | Zhou, J; Yuen, KY | Nature Medicine | 2020 | 372 | 99.2(26) |
| 65 | Characterizing responses to CFTR-modulating drugs using rectal organoids derived from subjects with cystic fibrosis | Beekman, JM | Science Translational Medicine | 2016 | 371 | 47.36(79) |
| 66 | A Three-Dimensional Organoid Culture System Derived from Human Glioblastomas Recapitulates the Hypoxic Gradients and Cancer Stem Cell Heterogeneity of Tumors Found In Vivo | Rich, JN | Cancer Research | 2016 | 367 | 45.88(81) |
| 67 | Human iPSC-Derived Cerebral Organoids Model Cellular Features of Lissencephaly and Reveal Prolonged Mitosis of Outer Radial Glia | Bershteyn, M; Kriegstein, AR | Cell Stem Cell | 2017 | 363 | 51.86(76) |
| 68 | Dynamic Three-Dimensional Culture Methods Enhance Mesenchymal Stem Cell Properties and Increase Therapeutic Potential | Genever, PG | Tissue Engineering Part C-Methods | 2010 | 360 | 26.34(98) |
| 69 | Organoid single-cell genomic atlas uncovers human-specific features of brain development | He, ZS; Treutlein, B; Camp, JG | Nature | 2019 | 358 | 79.56(41) |
| 70 | Trophoblast organoids as a model for maternal-fetal interactions during human placentation | Turco, MY; Moffett, A | Nature | 2018 | 358 | 67.13(54) |
| 71 | Patient-Derived Organoids Predict Chemoradiation Responses of Locally Advanced Rectal Cancer | Zhang, Z | Cell Stem Cell | 2020 | 357 | 84(37) |
| 72 | Synthetic hydrogels for human intestinal organoid generation and colonic wound repair | García, AJ | Nature Cell Biology | 2017 | 353 | 55.01(71) |
| 73 | Homeostatic mini-intestines through scaffold-guided organoid morphogenesis | Lutolf, MP | Nature | 2020 | 343 | 95.72(29) |
| 74 | Microglia innately develop within cerebral organoids | de Witte, LD | Nature Communications | 2018 | 342 | 62.18(62) |
| 75 | Paper-supported 3D cell culture for tissue-based bioassays | Whitesides, GM | PANS | 2009 | 340 | 23.58(99) |
| 76 | A dynamic multi-organ-chip for long-term cultivation and substance testing proven by 3D human liver and skin tissue co-culture | Wagner, I | Lab On A Chip | 2013 | 335 | 29.56(96) |
| 77 | Stress relaxing hyaluronic acid-collagen hydrogels promote cell spreading, fiber remodeling, and focal adhesion formation in 3D cell culture | Xia, Y | Biomaterials | 2018 | 333 | 54(73) |
| 78 | Differential Modulation by Akkermansia muciniphila and Faecalibacterium prausnitzii of Host Peripheral Lipid Metabolism and Histone Acetylation in Mouse Gut Organoids | Roeselers, G | Mbio | 2014 | 333 | 34.15(90) |
| 79 | Comparative Analysis and Refinement of Human PSC-Derived Kidney Organoid Differentiation with Single-Cell Transcriptomics | Humphreys, BD | Cell Stem Cell | 2018 | 332 | 62.25(61) |
| 80 | Identification of SARS-CoV-2 inhibitors using lung and colonic organoids | Pan, FC; Evans, T; Chen, SB | Nature | 2021 | 330 | 101.54(24) |
| 81 | Two Distinct Populations of Exosomes Are Released from LIM1863 Colon Carcinoma Cell-derived Organoids | Simpson, RJ | Molecular & Cellular Proteomics | 2013 | 329 | 29.68(95) |
| 82 | SARS-CoV-2 targets neurons of 3D human brain organoids | Gopalakrishnan, J | Embo Journal | 2020 | 324 | 92.57(31) |
| 83 | SARS-CoV-2 Infects the Brain Choroid Plexus and Disrupts the Blood-CSF Barrier in Human Brain Organoids | Lancaster, MA | Cell Stem Cell | 2020 | 320 | 96(27) |
| 84 | Preserved genetic diversity in organoids cultured from biopsies of human colorectal cancer metastases | Clevers, H | PANS | 2015 | 317 | 37.29(87) |
| 85 | Biophysically Defined and Cytocompatible Covalently Adaptable Networks as Viscoelastic 3D Cell Culture Systems | Anseth, KS | Advanced Materials | 2014 | 317 | 31.18(93) |
| 86 | Generation of human vascularized brain organoids | Waldau, B | Neuroreport | 2018 | 315 | 53.24(75) |
| 87 | Establishing Cerebral Organoids as Models of Human-Specific Brain Evolution | Pollen, AA; Kriegstein, AR | Cell | 2019 | 312 | 60.39(65) |
| 88 | Functional screening in human cardiac organoids reveals a metabolic mechanism for cardiomyocyte cell cycle arrest | Porrello, ER; Hudson, JE | PANS | 2017 | 312 | 48(78) |
| 89 | Comparison of cancer cells in 2D vs 3D culture reveals differences in AKT-mTOR-S6K signaling and drug responses | Dolznig, H | Journal of Cell Science | 2017 | 312 | 43.03(84) |
| 90 | Fabrication and Characterization of Magnetic Microrobots for Three-Dimensional Cell Culture and Targeted Transportation | Nelson, BJ | Advanced Materials | 2013 | 311 | 29.86(94) |
| 91 | Cerebral organoids at the air-liquid interface generate diverse nerve tracts with functional output | Lancaster, MA | Nature Neuroscience | 2019 | 305 | 61(64) |
| 92 | Oncogenic transformation of diverse gastrointestinal tissues in primary organoid culture | Kuo, CJ | Nature Medicine | 2014 | 305 | 31.28(92) |
| 93 | Highly Efficient Self-Healable and Dual Responsive Cellulose-Based Hydrogels for Controlled Release and 3D Cell Culture | Zhang, LN | Advanced Functional Materials | 2017 | 303 | 46.62(80) |
| 94 | Self-organization and symmetry breaking in intestinal organoid development | Liberali, P | Nature | 2019 | 301 | 61.22(63) |
| 95 | The Notch and Wnt pathways regulate stemness and differentiation in human fallopian tube organoids | Meyer, TF | Nature Communications | 2015 | 296 | 35.52(88) |
| 96 | A rectal cancer organoid platform to study individual responses to chemoradiation | Sawyers, CL; Smith, JJ | Nature Medicine | 2019 | 282 | 62.67(59) |
| 97 | Photoreceptor precursors derived from three-dimensional embryonic stem cell cultures integrate and mature within adult degenerate retina | Ali, RR | Nature Biotechnology | 2013 | 282 | 26.44(97) |
| 98 | Generation of human brain region-specific organoids using a miniaturized spinning bioreactor | Ming, GL | Nature Protocols | 2018 | 279 | 45.86(82) |
| 99 | Use of CRISPR-modified human stem cell organoids to study the origin of mutational signatures in cancer | Clevers, H | Science | 2017 | 279 | 42.92(85) |
| 100 | In vitro Toxicity Testing of Nanoparticles in 3D Cell Culture | Kotov, NA | Small | 2009 | 278 | 18.64(100) |
