# Supplementary material for: Interpretation of the past, present, and future of organoid technology: an updated bibliometric analysis from 2009 to 2024
Source: Front Cell Dev Biol. 2024 Aug 13;12:1433111. doi: 10.3389/fcell.2024.1433111 (PMC11347291; doi:10.3389/fcell.2024.1433111)
Supplement: Supplementary file 2 [file Table2.DOCX]

**Supplementary Table 2.** The 94 papers published in journals with a TPR>10% in organoids since 2021.

| **Rank** | **Title** | **Journal** | **Year** | **Total citations** |
| --- | --- | --- | --- | --- |
| 1 | Closing in on cancer heterogeneity with organoids | Nature Methods | 2024 | 0 |
| 2 | Glia-enriched cortical organoids implanted in mice capture astrocyte diversity | Nature Biotechnology | 2024 | 0 |
| 3 | Minimally invasive derivation of primary human epithelial organoids from fetal fluids | Nature Medicine | 2024 | 0 |
| 4 | Single-cell guided prenatal derivation of primary fetal epithelial organoids from human amniotic and tracheal fluids | Nature Medicine | 2024 | 0 |
| 5 | Morphological diversification and functional maturation of human astrocytes in glia-enriched cortical organoid transplanted in mouse brain | Nature Biotechnology | 2024 | 1 |
| 6 | Generation of complex bone marrow organoids from human induced pluripotent stem cells | Nature Methods | 2024 | 0 |
| 7 | Human conjunctiva organoids to study ocular surface homeostasis and disease | Cell Stem Cell | 2024 | 0 |
| 8 | Human fetal brain self-organizes into long-term expanding organoids | Cell | 2024 | 1 |
| 9 | Eavesdropping on brain organoids | Nature Biotechnology | 2024 | 0 |
| 10 | Kirigami electronics for long-term electrophysiological recording of human neural organoids and assembloids | Nature Biotechnology | 2024 | 1 |
| 11 | Human cerebellar organoids with functional Purkinje cells | Cell Stem Cell | 2024 | 2 |
| 12 | A pancreatic cancer organoid platform identifies an inhibitor specific to mutant KRAS | Cell Stem Cell | 2024 | 0 |
| 13 | Kidney organoid models reveal cilium-autophagy metabolic axis as a therapeutic target for PKD both in vitro and in vivo | Cell Stem Cell | 2024 | 0 |
| 14 | Safety and stable survival of stem-cell-derived retinal organoid for 2 years in patients with retinitis pigmentosa | Cell Stem Cell | 2023 | 3 |
| 15 | Trellis tree-based analysis reveals stromal regulation of patient-derived organoid drug responses | Cell | 2023 | 3 |
| 16 | Joint epigenome profiling reveals cell-type-specific gene regulatory programmes in human cortical organoids | Nature Cell Biology | 2023 | 0 |
| 17 | Joint epigenome profiling reveals cell-type-specific gene regulatory programmes in human cortical organoids | Nature Cell Biology | 2023 | 0 |
| 18 | FOXO inhibition rescues α- defensin expression in human intestinal organoids | PNAS | 2023 | 0 |
| 19 | Development of functional resident macrophages in human pluripotent stem cell-derived colonic organoids and human fetal colon | Cell Stem Cell | 2023 | 1 |
| 20 | iPS-cell-derived microglia promote brain organoid maturation via cholesterol transfer | Nature | 2023 | 8 |
| 21 | Tissue morphology influences the temporal program of human brain organoid development | Cell Stem Cell | 2023 | 2 |
| 22 | Complement factor D targeting protects endotheliopathy in organoid and monkey models of COVID-19 | Cell Stem Cell | 2023 | 2 |
| 23 | Spatiotemporal, optogenetic control of gene expression in organoids | Nature Methods | 2023 | 4 |
| 24 | Single-cell brain organoid screening identifies developmental defects in autism | Nature | 2023 | 10 |
| 25 | The mechano- chemical circuit drives skin organoid self-organization | PNAS | 2023 | 0 |
| 26 | Generating high-fidelity cochlear organoids from human pluripotent stem cells | Cell Stem Cell | 2023 | 6 |
| 27 | A single-cell multiomic analysis of kidney organoid differentiation | PNAS | 2023 | 5 |
| 28 | An in vivo neuroimmune organoid model to study human microglia phenotypes | Cell | 2023 | 27 |
| 29 | Multimodal spatiotemporal phenotyping of human retinal organoid development | Nature Biotechnology | 2023 | 9 |
| 30 | Generation of ventralized human thalamic organoids with thalamic reticular nucleus | Cell Stem Cell | 2023 | 4 |
| 31 | Stomach-derived human insulin-secreting organoids restore glucose homeostasis | Nature Cell Biology | 2023 | 5 |
| 32 | Human airway and nasal organoids reveal escalating replicative fitness of SARS-CoV-2 emerging variants | PNAS | 2023 | 21 |
| 33 | A multi-organoid platform identifies CIART as a key factor for SARS-CoV-2 infection | Nature Cell Biology | 2023 | 8 |
| 34 | Engineered human hepatocyte organoids enable CRISPR-based target discovery and drug screening for steatosis | Nature Biotechnology | 2023 | 33 |
| 35 | Tubuloid culture enables long-term expansion of functional human kidney tubule epithelium from iPSC-derived organoids | PNAS | 2023 | 4 |
| 36 | Controlling organoid symmetry breaking uncovers an excitable system underlying human axial elongation | Cell | 2023 | 18 |
| 37 | Structural and functional integration of human forebrain organoids with the injured adult rat visual system | Cell Stem Cell | 2023 | 24 |
| 38 | Controlling human organoid symmetry breaking reveals signaling gradients drive segmentation clock waves | Cell | 2023 | 17 |
| 39 | In vivo development of immune tissue in human intestinal organoids transplanted into humanized mice | Nature Biotechnology | 2023 | 21 |
| 40 | Re-formation of synaptic connectivity in dissociated human stem cell-derived retinal organoid cultures | PNAS | 2023 | 8 |
| 41 | Organoid modeling of human fetal lung alveolar development reveals mechanisms of cell fate patterning and neonatal respiratory disease | Cell Stem Cell | 2023 | 19 |
| 42 | Mapping prohormone processing by proteases in human enteroendocrine cells using genetically engineered organoid models | PNAS | 2022 | 3 |
| 43 | Bovine and human endometrium-derived hydrogels support organoid culture from healthy and cancerous tissues | PNAS | 2022 | 18 |
| 44 | En masse organoid phenotyping informs metabolic-associated to NASH | Cell | 2022 | 24 |
| 45 | Geometric engineering of organoid culture for enhanced organogenesis in a dish | Nature Methods | 2022 | 17 |
| 46 | Human induced pluripotent stem cell-derived salivary gland organoids model SARS-CoV-2 infection and replication | Nature Cell Biology | 2022 | 15 |
| 47 | Maturation and circuit integration of transplanted human cortical organoids | Nature | 2022 | 100 |
| 48 | Inferring and perturbing cell fate regulomes in human brain organoids | Nature | 2023 | 50 |
| 49 | Proper acquisition of cell class identity in organoids allows definition of fate specification programs of the human cerebral cortex | Cell | 2022 | 34 |
| 50 | De novo construction of T cell compartment in humanized mice engrafted with iPSC-derived thymus organoids | Nature Methods | 2022 | 15 |
| 51 | Optimized human intestinal organoid model reveals interleukin-22-dependency of paneth cell formation | Cell Stem Cell | 2022 | 34 |
| 52 | Human ureteric bud organoids recapitulate branching morphogenesis and differentiate into functional collecting duct cell types | Nature Biotechnology | 2023 | 26 |
| 53 | Development and characterization of human fetal female reproductive tract organoids to understand Mullerian duct anomalies | PNAS | 2022 | 8 |
| 54 | Uncovering the mode of action of engineered T cells in patient cancer organoids | Nature Biotechnology | 2023 | 44 |
| 55 | A scalable organoid model of human autosomal dominant polycystic kidney disease for disease mechanism and drug discovery | Cell Stem Cell | 2022 | 28 |
| 56 | Automated high-speed 3D imaging of organoid cultures with multi-scale phenotypic quantification | Nature Methods | 2022 | 24 |
| 57 | Enhanced cortical neural stem cell identity through short SMAD and WNT inhibition in human cerebral organoids facilitates emergence of outer radial glial cells | Nature Cell Biology | 2022 | 19 |
| 58 | Stem-cell-derived trophoblast organoids model human placental development and susceptibility to emerging pathogens | Cell Stem Cell | 2022 | 49 |
| 59 | Human branching cholangiocyte organoids functional bile duct formation | Cell Stem Cell | 2022 | 15 |
| 60 | Generation of 3D lacrimal gland organoids from human pluripotent stem cells | Nature | 2022 | 17 |
| 61 | Human organoid models to study SARS-CoV-2 infection | Nature Methods | 2022 | 58 |
| 62 | 3D-organoid culture supports differentiation of human CAR+ iPSCs into highly functional CAR T cells | Cell Stem Cell | 2022 | 53 |
| 63 | Bisphenol A replacement chemicals, BPF and BPS, induce protumorigenic changes in human mammary gland organoid morphology and proteome | PNAS | 2022 | 18 |
| 64 | Cone photoreceptors in human stem cell-derived retinal organoids demonstrate intrinsic light responses that mimic those of primate fovea | Cell Stem Cell | 2022 | 23 |
| 65 | Deterministic scRNA-seq captures variation in intestinal crypt and organoid composition | Nature Methods | 2022 | 27 |
| 66 | SARS-CoV-2 infects the human kidney and drives fibrosis in kidney organoids | Cell Stem Cell | 2022 | 127 |
| 67 | Androgens increase excitatory neurogenic potential in human brain organoids | Nature | 2022 | 29 |
| 68 | Functional human gastrointestinal organoids can be engineered from three primary germ layers derived separately from pluripotent stem cells | Cell Stem Cell | 2022 | 41 |
| 69 | Genome-wide screening in human kidney organoids identifies developmental and disease-related aspects of nephrogenesis | Cell Stem Cell | 2022 | 28 |
| 70 | Circadian key component CLOCK/BMAL1 interferes with segmentation clock in mouse embryonic organoids | PNAS | 2022 | 11 |
| 71 | Lineage recording in human cerebral organoids | Nature Methods | 2022 | 65 |
| 72 | Adult mouse and human organoids derived from thyroid follicular cells and modeling of Graves' hyperthyroidism | PNAS | 2021 | 17 |
| 73 | Human microglia states are conserved across experimental models and regulate neural stem cell responses in chimeric organoids | Cell Stem Cell | 2021 | 83 |
| 74 | Co-emergence of cardiac and gut tissues promotes cardiomyocyte maturation within human iPSC-derived organoids | Cell Stem Cell | 2021 | 53 |
| 75 | Human brain organoids assemble functionally integrated bilateral optic vesicles | Cell Stem Cell | 2021 | 60 |
| 76 | Culturing patient-derived malignant hematopoietic stem cells in engineered and fully humanized 3D niches | PNAS | 2021 | 19 |
| 77 | Generation of hypothalamic arcuate organoids from human induced pluripotent stem cells | Cell Stem Cell | 2021 | 54 |
| 78 | Inflation-collapse dynamics drive patterning and morphogenesis in intestinal organoids | Cell Stem Cell | 2021 | 35 |
| 79 | ELAVL4, splicing, and glutamatergic dysfunction precede neuron loss in MAPT mutation cerebral organoids | Cell | 2021 | 45 |
| 80 | Organoid modeling of Zika and herpes simplex virus 1 infections reveals virus-specific responses leading to microcephaly | Cell Stem Cell | 2021 | 54 |
| 81 | Patient-derived organoids model cervical tissue dynamics and viral oncogenesis in cervical cancer | Cell Stem Cell | 2021 | 80 |
| 82 | Exploring the human lacrimal gland using organoids and single-cell sequencing | Cell Stem Cell | 2021 | 49 |
| 83 | Mechanical compartmentalization of the intestinal organoid enables crypt folding and collective cell migration | Nature Cell Biology | 2021 | 78 |
| 84 | Charting human development using a multi-endodermal organ atlas and organoid models | Cell | 2021 | 52 |
| 85 | Modeling plasticity and dysplasia of pancreatic ductal organoids derived from human pluripotent stem cells | Cell Stem Cell | 2021 | 54 |
| 86 | Resolving organoid brain region identities by mapping single-cell genomic data to reference atlases | Cell Stem Cell | 2021 | 40 |
| 87 | Commitment and oncogene-induced plasticity of human stem cell-derived pancreatic acinar and ductal organoids | Cell Stem Cell | 2021 | 48 |
| 88 | Capture and metabolomic analysis of the human endometrial epithelial organoid secretome | PNAS | 2021 | 26 |
| 89 | Plasticity of distal nephron epithelia from human kidney organoids enables the induction of ureteric tip and stalk | Cell Stem Cell | 2021 | 60 |
| 90 | Quantifying single-cell ERK dynamics in colorectal cancer organoids reveals EGFR as an amplifier of oncogenic MAPK pathway signalling | Nature Cell Biology | 2021 | 59 |
| 91 | An organoid-based organ-repurposing approach to treat short bowel syndrome | Nature | 2021 | 83 |
| 92 | Human heart-forming organoids recapitulate early heart and foregut development | Nature Biotechnology | 2021 | 172 |
| 93 | Modeling human adaptive immune responses with tonsil organoids | Nature Medicine | 2021 | 101 |
| 94 | Volumetric Compression Induces Intracellular Crowding to Control Intestinal Organoid Growth via Wnt/β-Catenin Signaling | Cell Stem Cell | 2021 | 53 |
